# Dimethyl fumarate combined with cisplatin at subcytotoxic doses sensitizes cervical cancer toward ferroptosis and apoptosis through GSH restriction and p53 (re)activation

**DOI:** 10.1002/1878-0261.70216

**Published:** 2026-03-25

**Authors:** Carolina Punziano, Giuseppina Minopoli, Simona Romano, Laura Marrone, Katia Aquilano, Maria Lina Tornesello, Raffaella Faraonio

**Affiliations:** ^1^ Department of Molecular Medicine and Medical Biotechnology University of Naples Federico II Italy; ^2^ Department of Biology University of Rome Tor Vergata Italy; ^3^ Molecular Biology and Viral Oncology Unit Istituto Nazionale Tumori IRCCS Fondazione G. Pascale Naples Italy

**Keywords:** apoptosis, cervical cancer, dimethyl fumarate, ferroptosis, organoids, TP53

## Abstract

Cervical cancer is one of the leading causes of tumor‐related deaths among women. Chemotherapy in cervical cancer is mainly based on cisplatin, but this drug has limited efficacy; therefore, alternative treatment options are needed. Ferroptosis represents a novel form of cell death. In cervical epithelium, ferroptosis occurs in the early neoplastic stages of papillomavirus infection but shifts to evasion in carcinoma. Combination therapy has the potential to enhance cancer cell death and overcome resistance development. Herein we demonstrate that dimethyl fumarate (DMF), a Food and Drug Administration (FDA)‐approved anti‐inflammatory drug, induces ferroptosis in cervical cancer cells in a dose‐dependent manner and inhibits growth in spheroid models. Cotreatment with DMF and cisplatin significantly decreases cell viability compared to either drug alone. Under DMF/cisplatin combination, cervical cancer cells underwent to glutathione depletion and p53 (re)activation, leading to cell death by both ferroptosis and apoptosis. We found a p53‐mediated downregulation of the Solute Carrier Family 7 Member 11 (SLC7A11)/Cystine/Glutamate Transporter (xCT) expression and glutathione levels. Our results suggest that combined administration of DMF and cisplatin, by targeting the dependency of cervical cancer cells on glutathione and (re)activating p53, represents a promising anticancer therapeutic strategy.

## Graphical abstract

Dimethyl fumarate (DMF) reduces growth of HPV‐positive cervical cancer spheroids and induces ferroptosis in cervical cancer cells via blocking SLC7A11/Glutathione (GSH) axis. Combination of subcytotoxic doses of DMF and cisplatin (CDDP) further suppresses spheroid growth and drives cell death in 2D culture models. Mechanistically, DMF plus CDDP by reducing the SLC7A11/GSH axis via p53 (re)activation, promotes both ferroptosis and apoptosis.

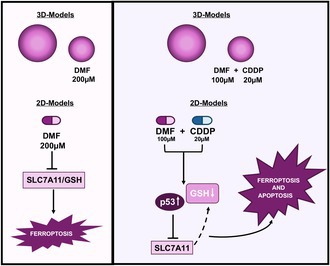

**Abbreviations**
ATF3: activating transcription factor 3
ATF4: activating transcription factor 4
BSO: buthionine sulfoximine
CC: cervical cancer
CDDP: cisplatin
CDKN1A/p21: cyclin‐dependent kinase inhibitor 1A
CHAC1: glutathione‐specific gamma‐glutamylcyclotransferase1
DMF: dimethyl fumarate
DMSO: dimethyl sulfoxide
E6AP/UBE3A: E6‐associated protein / ubiquitin protein ligase E3A
ER: erastin
FER‐1: ferrostatin‐1
GADD45α: growth arrest and DNA damage‐inducible 45 alpha
GCLM: glutamate–cysteine ligase modifier subunit
GPX4: glutathione peroxidase 4
GSH: glutathione
HR‐HPVs: high‐risk human papillomaviruses
hTERT: human telomerase reverse transcriptase
KRAS: Kirsten rat sarcoma viral oncogene homolog
LAP2: lamina‐associated polypeptide 2
MDA: malondialdehyde
Nrf2: nuclear factor erythroid 2–related factor 2
p27/CDKN1B: cyclin‐dependent kinase inhibitor 1B
p53: tumor protein p53
PARP‐1: poly (ADP‐ribose) polymerase 1
pRB: retinoblastoma protein
PTGS2: prostaglandin‐endoperoxide synthase 2
ROS: reactive oxygen species
RSL3: RAS‐selective lethal 3
SAS: sulfasalazine
SAT‐1: spermidine/spermine N1‐acetyltransferase 1
SIL: squamous intraepithelial lesion
SLC3A2: solute carrier family 3 member 2
SLC7A11: solute carrier family 7 member 11
SOCS1: suppressor of cytokine signaling 1
STAT3: signal transducer and activator of transcription 3
xCT: cystine/glutamate transporter

## 1 Introduction

Cervical cancer represents one of the leading causes of tumor‐related deaths among women, worldwide [1, 2]. The primary etiological factor in cervical cancer development is persistent infection with high‐risk human papillomaviruses (HR‐HPVs) [3, 4]. Although preventive vaccinations against HPV infection are available, an increase in the rate of cervical carcinoma has been reported in the last decades [5]. Studies conducted over the past 30 years have shown that the viral proteins E5, E6, and E7 are crucial drivers of HPV‐mediated transformation [6]. They interact with and inactivate several proteins of the host cells involved in signaling cascades essential for proper cycle regulation and tumor suppression [6, 7, 8, 9]. In addition, E6 and E7 proteins act in concert to induce tumors in transgenic mice [10, 11, 12, 13]. The oncoprotein E6 enhances p53 degradation via the ubiquitin‐proteasome pathway by interacting with the cellular ubiquitin ligase E6AP/UBE3A (E6‐associated protein) [14]. Consequently, abrogation of p53 tumor suppressor function causes genomic instability and impairs apoptosis in response to DNA damage. Furthermore, the E6 oncoprotein fosters human telomerase reverse transcriptase (hTERT) expression in a p53‐independent axis [15, 16, 17]. The major function of the E7 oncoprotein is the negative control of the retinoblastoma protein (pRb) [18], leading to the release of E2F transcription factors which promote cell cycle progression [7]. E7 can also interact with other cell cycle regulatory proteins, such as cyclin‐dependent kinase inhibitors (e.g., CDKN1A/p21 and CDKN1B/p27), thereby accelerating cell cycle progression and contributing to malignant transformation [9].

Ferroptosis is a programmed cell death distinct from apoptosis and other forms of death [19]. It is characterized by the accumulation of toxic lipid peroxides, produced through dioxygenation, due to dysfunction in antioxidant systems such as glutathione (GSH), increased reactive oxygen species (ROS), and iron accumulation. These changes cause irreversible damage to the cell membrane and ultimately cell death [20].

Data over the past 20 years indicate that ferroptosis can contribute to the progression of various diseases or even prevent certain pathophysiological processes [21, 22, 23]. In fact, on one hand, ferroptosis has been causally linked to neurodegenerative disorders [24], blood diseases [25], senescence/aging [26], as well as to kidney‐ and ischemia–reperfusion injuries [27, 28]; on the other hand, ferroptosis is considered a tumor suppressor mechanism, and its induction represents a novel therapeutic option to inhibit the growth of different tumor types [23, 29, 30]. Unlike other types of cell death, ferroptosis holds significant potential in translational medicine, particularly in overcoming therapy resistance in cancer. Pharmacological activators of ferroptosis have been proposed as potential therapeutic agents to promote cancer cell death [31].

Several studies have demonstrated that ferroptosis is a key mechanism in cervical carcinogenesis and treatment response, as reviewed in Refs [32, 33]. Specifically, it has been reported that in HPV‐infected cervical cancer cells, ferroptosis occurs during the transition from normal to squamous intraepithelial lesion (SIL) [34]. However, during the transformation from SIL to squamous carcinoma, some cells acquire anti‐ferroptosis mechanisms mainly driven by KRAS signaling that facilitate both survival and cervical cancer progression. Under ferroptosis‐inducing stimuli, HPV‐positive cervical cancer cells can increase activities of Glutamate–Cysteine Ligase Modifier Subunit (GCLM) and Glutathione Peroxidase 4 (GPX4) to activate lipid peroxidation clearance, which correlates with *in vivo* expression data [34]. Therefore, it is essential to identify novel drugs, treatments, or synergistic therapies that sensitize cervical cancer cells to ferroptosis, thereby enhancing responses to conventional treatments and preventing tumor recurrence.

GSH is an endogenous antioxidant molecule and essential component of defenses against ferroptosis, being a cofactor of the GPX4 enzyme that repairs lipid peroxides [19, 35, 36]. Accordingly, a metabolomic analysis revealed that cysteine and glutathione increase in cervical cell lines [37]. Preservation of intracellular GSH consequently improves cell survival, whereas its loss predisposes cells to death. GSH levels are sustained by the activity of the trans‐membrane x_c_‐ system composed of the Solute Carrier Family 7 Member 11 (SLC7A11)/Cystine/Glutamate Transporter (xCT) and the Solute Carrier Family 3 Member 2 (SLC3A2)/4F2 Cell‐Surface Antigen Heavy Chain (4F2hc) subunits, that exchanges cystine (the precursor of cysteine) into cells with glutamate export [19, 38, 39]. Concerning SLC7A11, it has been demonstrated that following its inhibition, cancer cells become more sensitive to radiotherapy [40].

Cisplatin‐based first‐line chemotherapy shows limited efficacy in cervical cancer [41]. Additionally, its clinical use is often restricted by adverse effects and toxicities affecting the gastrointestinal, renal, neurological, and hematological systems [42]. Therefore, there is a need to identify new treatment options for cervical cancer. Combination therapies, which employ drugs with different mechanisms of action, have the potential to eliminate a greater number of cancer cells and overcome resistance, provided effective drug combinations are identified (reviewed in Ref. [43]). Dimethyl fumarate (DMF) is an FDA‐approved anti‐inflammatory drug, and emerging studies suggest that DMF also exerts an antitumor activity in some types of cancers, including glioblastoma, melanoma, and colon cancer [44, 45, 46].

In the present study, we aimed to evaluate the antitumor efficacy of DMF in cervical cancer. Specifically, we investigated whether high doses of DMF (200 μm) can induce ferroptosis in 2D cervical cancer cell models and reduce growth in 3D spheroid systems. We also explored whether subcytotoxic DMF, in combination with cisplatin doses below the IC50, enhances cancer cell death by promoting both ferroptosis and apoptosis pathways. Finally, we examined the involvement of glutathione‐dependent mechanisms, including the Nrf2‐SLC7A11 axis and p53 signaling, in mediating the cellular response to DMF and cisplatin.

## 2 Materials and methods

### 2.1 Cell cultures and reagents

SiHa (RRID: CVCL_0032), C4I (RRID: CVCL_2253), HeLa (RRID: CVCL_0030), and Caski (RRID: CVCL_1100) cervical cancer cell lines were purchased from the American Type Culture Collection (ATCC, Manassas, VA, USA). Cell lines used in this research were authenticated by short tandem repeat (STR) profiling within the past 3 years (July 2025). All experiments were conducted using mycoplasma‐free cells, confirmed through PCR testing. SiHa, C4I, and HeLa cells were grown in Dulbecco's modified Eagle's medium (DMEM, #PMSTVMSV; Microgem, Naples, Italy), Caski cells in Roswell Park Memorial Institute 1640 (RPMI, #31870‐025; Thermo Fisher Scientific, Monza, Italy) Medium, all supplemented with 10% fetal bovine serum (FBS, Low Endotoxin South American Origin # RM10432; Microgem) and 1% of an antibiotic's mixture (penicillin and streptomycin # PBSAVSMV; Microgem) at 37 °C and 5% CO_2_. All the treatments were performed on cells at 70% confluence, seeded 24 h prior to start treatments. Dimethyl fumarate (#242926; Sigma‐Aldrich, St. Louis, MO, USA), sulfasalazine (#S0883; Sigma‐Aldrich), dimethyl sulfoxide (DMSO) (#A3672; Applichem, Darmstadt, Germany), cisplatin was provided by clinicians at 1 mg·mL^−1^ (Accord Healthcare, Harrow, UK) protocols of Policlinico Federico II (Naples, Italy).

### 2.2 Cell viability assay

Cell viability was evaluated using the Cell Counting Kit‐8 (E‐CK‐A362; Elabscience Hustion, Texas, USA), according to the manufacturer's protocol. Briefly, cells were seeded in 96‐well plates at a density of 5.5 × 10^3^ cells the day before treatments. After treatments, the cells were incubated with fresh complete medium containing 10% (v/v) of CCK‐8 buffer provided in the kit for 1 h. The absorbance was measured at 450 nm using a Synergy H1 Hybrid multiplate reader (Agilent Bio Tek, Santa Clara, CA, USA). The relative cell viability was calculated by normalizing the absorbance of treated cells to that of control cells treated with vehicle.

### 2.3 Generation of 3D models

3D models from SiHa and C4I cells were generated by combining Hanging drop method and the use of methylcellulose (MC) (#M0512; Sigma‐Aldrich) in the media, as described by Ware et al. [47] to limit their disaggregation. Briefly, cells were counted and resuspended into an adequate volume of complete culture medium containing 10% of MC to achieve a final concentration of 500 cells·μL^−1^. Drops of 20 μL were pipetted onto the lid of 100 mm dishes that were then inverted over dishes containing 10 mL phosphate buffer solution (PBS, #TL1006; Microgem) to maintain humidity. Hanging drops were cultured at 37 °C and 5% CO_2_ for 4 days. The resultant cell aggregates (spheroids) were transferred into 96‐well plates with round bottom (#MS‐9096UZ; S‐BIO, Tokyo, Japan) containing 100 μL of complete culture medium. Subsequently, spheroids were treated as specified, and the images were acquired using an inverted light microscope (Leica, Wetzlar, Germany). To analyze the size of spheroids, the imagej software (NIH, Bethesda, MD, USA) was used. The area of each spheroid was determined by manually drawing a line around its perimeter.

### 2.4 Cell growth assay

To assess cell proliferation over time, cell growth assays were performed by manual cell counting. SiHa and C4I cells were seeded at a density of 7.5 × 10^4^ cells/well in 24‐well plates containing complete DMEM medium. Twenty four hours after seeding, cells were treated with DMF (100, 150 and 200 μm) or vehicle. At 24, 48, and 72‐h post‐treatment, cells were collected by trypsinization and resuspended in PBS. The number of cells was counted using a Bürker hemocytometer under a light microscope (Leica). Data are expressed as cell number per milliliter.

### 2.5 Malondialdehyde (MDA) assay

The MDA amounts were measured using a colorimetric assay kit (E‐BC‐K028‐M; Elabscience Biotechnology). After treatments, 3 × 10^6^ cells were washed with PBS and lysed in 500 μL of the supplied extraction solution using ultrasonic cell disruptor. To remove insoluble material, a centrifugation at 10 000 ***g*** for 10 min at 4 °C was performed. Subsequently, 0.1 mL of lysate samples were mixed with 1 mL of working solution (supplied by kit) and incubate at 100 °C for 40 min. After incubation, the materials were centrifuged at 1078 ***g*** for 10 min at 4 °C. Finally, 250 μL of the supernatant were transferred into 96‐well plate and the OD in each well was measured at 532 nm using Synergy H1 Hybrid multiplate reader (Agilent Bio Tek).

### 2.6 Glutathione colorimetric detection assay

To determine the GSH amounts, a glutathione colorimetric detection kit (# EIAGSHC; Invitrogen, Carlsbad, CA, USA) was used. Briefly, upon treatments, 5.5 × 10^3^ SiHa and C4I cells (5 × 10^5^) were lysed in 75 μL of 5% salicylic acid provided in the kit and then centrifugated at 21 953 ***g*** for 10 min at 4 °C. The supernatants were collected for the assays. For the quantization, samples (50 μL) were mixed with equal volumes of supplied colorimetric detection reagent and reaction mixture (25 μL each) in a supplied half‐area 96‐well plate and incubated for 20 min at room temperature. Then, the absorbance at 405 nm was measured using Synergy H1 Hybrid multiplate reader (Agilent Bio Tek).

### 2.7 Lipid peroxidation assay

Lipid peroxidation was measured using the Image‐iT Lipid Peroxidation Kit (# C10445; Invitrogen), based on the BODIPY 581/591 C11 fluorescent probe, which shifts emission from red to green upon oxidation. Briefly, SiHa and C4I cells were seeded in plated on μ‐Slide 8 wells (#80826; ibidi GmnH, Grafelfing, Germany) 24 h prior to treatments. According to the manufacturer's instructions, treated and untreated cells were washed twice in PBS and incubated into the complete growth medium with 10 μm Image‐iT Lipid Peroxidation Sensor for 30 min at 37 °C. Hoechst 33342 (Bio‐Rad, Hercules, CA, USA) was added during the remaining 15 min incubation to stain the nucleus of live cells. Pictures were captured by using the Leica Thunder Imaging System (Leica Microsystems, Wetzlar, Germany) incorporating a Lumencor fluorescence LED light source and a LEICA DFC9000 GTC camera. Images were captured with las x software (Leica Microsystems). Z‐slice images were acquired using 63× oil immersion objectives.

### 2.8 Click‐iT™ EdU Alexa Fluor™ 488 flow cytometry assay

Proliferation of SiHa and C4I cells was assessed using the Click‐iT™ EdU Alexa Fluor™ 488 Flow Cytometry Assay Kit (Thermo Fisher Scientific), according to the manufacturer's instructions. Briefly, 7.5 × 10^4^ cells were incubated with 15 μm 5‐ethynyl‐2′‐deoxyuridine (EdU) for 2 h. After incubation, cells were harvested, washed with phosphate‐buffered saline (PBS) fixed and permeabilized using the fixative and saponin‐based buffers provided in the kit. Finally, bulk DNA was stained using propidium iodide (PI) dye. Samples were analyzed by flow cytometry by using a MACSQuant Analyzer 10 (Miltenyi Biotec, Gladbach, NWR, Germany), and data were analyzed using flowjo software (Pharmingen/Becton Dickinson, Ashland, OR, USA).

### 2.9 Analysis of cell death

Cell death was measured using propidium iodide (PI) in double staining with annexin V‐FITC. Briefly, 7.5 × 10^4^ SiHa and C4I cells were treated with DMF (100 or 200 μm) in absence or in presence of Ferrostatin‐1 (10 μm) and harvested at 48 or at 72 h. Cell pellets were resuspended in 100 μL of binding buffer (10 μm Hepes/NaOH pH 7.5, 140 μm NaCl, and 2.5 μm CaCl_2_) containing 1 μL of annexin‐V‐FITC (Pharmingen/Becton Dickinson, San Diego, CA, USA) and 10 μL of PI (Pharmingen/Becton Dickinson) and incubated for 15 min at room temperature in the dark. Flow cytometry acquisition was performed using a MACSQuant Analyzer 10 (Miltenyi Biotec), and data were analyzed using flowjo software (Pharmingen/Becton Dickinson).

### 2.10 RNA extraction and real‐time quantitative PCR

Total RNAs were extracted using the TRIzol reagent (#15596018; Ambion, Life Technologies, Carlsbad, CA, USA). SiHa and C4I cells (1 × 10^6^) were resuspended in 500 μL of TRIzol reagent and incubated for 5 min at room temperature. Then, 100 μL of chloroform was added to the lysates, which were centrifuged at 21.953 ***g*** for 20 min at 4 °C. The upper aqueous phase containing RNAs was transferred into new tubes, and RNAs were precipitated adding 250 μL of isopropanol and incubated at room temperature for 10 min. To recover RNAs, samples were centrifuged at 21.953 ***g*** for 20 min at 4 °C. The resulting RNA pellet was washed with 75% ethanol and then resuspended into RNase‐free water and incubated at 60 °C for 10 min. For real‐time quantitative PCR (RT‐qPCR), the synthesis of cDNAs was performed using the SensiFAST cDNA Synthesis Kit (#BIO‐65054; Bio‐Line‐Aurogene, Italy) following the manufacturer's instructions on the T100 Thermal Cycler (Bio‐Rad). Reactions occurred in a final volume of 20 μL, and aliquots of cDNAs were used in quantitative PCRs. The analyses were conducted on the CFX96 real‐time system instrument (Bio‐Rad) using SensiFAST SYBR No‐ROX (#BIO‐98020; Bio‐Line‐Aurogene, Rome, Italy). Beta 2‐microglobulin was used for internal normalization; primers used for RT‐qPCR analyses are reported in Table S1. Relative fold variations were calculated using the $2^{-\Delta \Delta C_{t}}$ method [48].

### 2.11 Proteins extraction and western blotting assay

#### 2.11.1 Total protein preparation

SiHa and C4I cells (5 × 10^5^) were lysed using the RIPA buffer (NaCl 150 mm, NP40 1%, sodium deoxycholate 0.5%, SDS 0.1%, Tris 50 mm, pH 7.5) supplemented with protease and phosphatase inhibitors (Sigma‐Aldrich). The lysates were centrifuged at 21.953 ***g*** for 30 min at 4 °C and supernatants were used for protein quantification by Bio‐Rad protein assay (Bio‐Rad).

#### 2.11.2 Nucleus/cytoplasm fractionation

SiHa and C4I cells (1 × 10^6^) were resuspended in 500 μL of hypotonic solution (10 mm Hepes, 1.5 mm MgCl_2_, 10 mm KCl 0.1% NP‐40) supplemented with protease and phosphatase inhibitors and homogenized using a 27‐gauge needle, next incubated on ice for 30 min. The suspension was then centrifuged at 1000 ***g*** for 10 min at 4 °C to pellet the nuclei, while the supernatant was collected as the cytoplasmic fraction. The pellet of nuclei was lysed utilizing 100 μL of nuclear lysis buffer (5 mm HEPES, 1.5 mm MgCl_2_, 300 mm NaCl, 0.1 mm EDTA, 52% glycerol) supplemented with protease and phosphatase inhibitors for 20 min on ice and then centrifugate at 18 000 ***g*** for 10 min. Protein quantification of nucleus and cytoplasm fractionations was obtained with Bio‐Rad protein assay (Bio‐Rad). Western blotting: Protein aliquots (usually 30/40 μg) were denatured at 95 °C for 5 min in a Laemmli sample buffer (200 mm Tris/HCL pH 6.8, 8% SDS, 40% glycerol and 0.005% Bromophenol Blue) containing 0.1 mm of DTT (#A2948,0005; Applichem) and separated on SDS/PAGE gels. Then, proteins were transferred to nitrocellulose membrane (#GE10600117; Amersham, Cytiva, Milan, Italy) at 100 V for 90 min. The membranes were first stained with Ponceau S (0.1% (w/v) in 5% acetic acid, #P3504; Sigma‐Aldrich) and then blocked with 5% nonfat dry milk in TBS (20 mm Tris/HCL pH 7.5 and 150 mm NaCl) containing 0.1% Tween 20% at room temperature for 45 min. ATF4 (sc‐390063, 1:500; Santa Cruz Biotechnology, Dallas, TX, USA) Caspase‐3 (E‐AB‐60646, 1 : 500; Elabscience), LAP2 (sc‐81610, 1 : 1000; Santa Cruz Biotechnology), Nrf2 (sc‐13032; 1 : 300; Santa Cruz Biotechnology) p53 (sc‐126, 1 : 1000; Santa Cruz Biotechnology), PARP‐1 (sc‐8007, 1 : 1000; Santa Cruz Biotechnology), Phospho‐p53 (Ser15) (#9284, 1 : 500; Cell Signaling, USA), Phospho‐STAT3 (#D3A7, 1 : 500; Cell Signaling), STAT3 (sc‐8019, 1 : 1000; Santa Cruz Biotechnology), and xCT/SLC7A11 (#D2M7A, 1 : 1000; Cell Signaling Technology, Danvers, MA, USA) antibodies were appropriately diluted in TBS‐tween and used for membrane incubation at 4 °C for overnight. After incubation with rabbit/mouse horseradish peroxidase‐conjugated secondary antibodies at room temperature for 45 min; the antigen–antibody complexes visualized using ECL chemiluminescence reagent kit (#EMP011005 LiteAblot® PLUS; Euroclone, Milan, Italy) and the Chemidoc instrument (Chemidoc; Bio‐Rad).

### 2.12 Caspase‐3/7 activity assay

Caspase‐3/7 activity was measured using the Caspase‐3/7 Activity Assay Kit (E‐CK‐A383; Elabscience) according to the manufacturer's instructions. Briefly, after treatments, cells were washed twice with cold PBS, lysed on ice using the supplied Cell Lysis Buff and centrifuged at 12 000 ***g*** for 10 min at 4 °C. For the enzymatic reaction, 50 μL of each sample, normalized for protein concentration using Bradford method assay (Bio‐Rad), was added to 96‐well plates, with 50 μL of 2× Reaction Buffer. The mixtures were incubated at 37 °C for 1 h, and absorbance was read at 405 nm using a Synergy H1 Hybrid multiplate reader (Agilent Bio Tek).

### 2.13 Statistical analyses

Statistical analyses were performed using GraphPad Prism (version 10.3.1 GraphPad Software LLC, San Diego, CA, USA). Statistical significance is denoted as follows: ns: *P*‐value of ≥ 0.05, *P < 0.05, **P < 0.01, ***P < 0.001, ****P < 0.0001.

## 3 Results

### 3.1 Effects of ferroptosis inducer compounds on cervical cancer cells

**Fig. 1:**
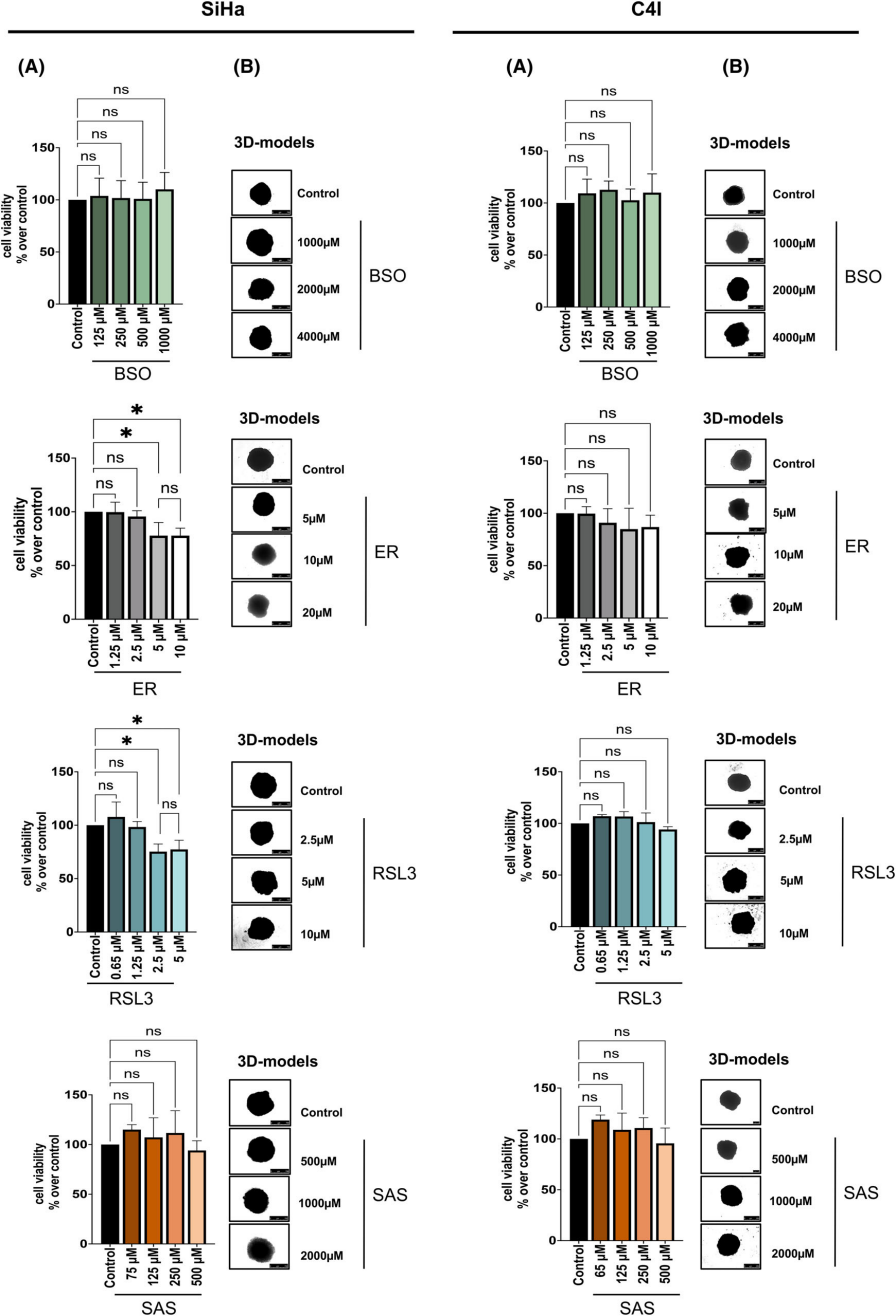
Effect of ferroptosis inducers on the growth of SiHa and C4I cells (2D and 3D models). (A) SiHa and C4I cells were treated with the indicated concentrations of drugs [Buthionine Sulfoximine (BSO), Erastin (ER), RAS‐selective lethal 3 (RSL3), or sulfasalazine (SAS)] for 24 h. Cell viability was assessed by CCK‐8 assay. Data are reported as relative percentages of optical density obtained in treated cells compared to cells treated with vehicle (control). Data represents means ± SD from three independent experiments (*n* = 3). Statistical significance was assessed by one‐way ANOVA: *P ≤ 0.01; ns, not statistically significant. (B) 3D spheroids obtained from SiHa and C4I cells (see Section 2) were exposed for 96 h to vehicle (control) or single doses of drugs as indicated. Representative images of 3D cultures were captured by inverted microscopy (scale bar: SiHa, 750 μm or C4I, 250 μm).

We evaluated the inhibitory effect of canonical inducers of ferroptosis on the proliferation of human cervical cancer‐derived cell lines SiHa and C4I, which contain HPV16 and HPV18 DNA, respectively. Specifically, we selected Buthionine sulfoximine (BSO), Erastin (ER), RAS‐selective lethal 3 (RSL3), and Sulfasalazine (SAS), known to induce ferroptosis in various tumor types [34, 49, 50, 51, 52]. Using Cell Counting Kit‐8 (CCK‐8) assays, we found that treatments with Erastin at 5 and 10 μm and RSL3 at 2.5 and 5 μm for 24 h caused a relatively modest but statistically significant decrease in viability of SiHa cells, compared to control groups; however, no differences in viability were observed at these drug concentrations (Fig. 1A). Viability of C4I cells was statistically unaffected by the above treatments, also at maximum drug concentrations (Fig. 1A). The other compounds BSO and SAS showed no effects on the viability of both cell types compared to control groups. In addition, we evaluated the effectiveness of these compounds in two other cervical cell lines, the Caski (harboring HPV16) and HeLa (infected with HPV18), using equal doses and treatment time. Data obtained by CCK‐8 assays show that all tested compounds do not affect cell viability of both Caski and HeLa cells (Fig. S1). These results suggested that the HPV‐positive cervical cancer cell models analyzed in this study are strongly resistant to diverse types of drugs inducing ferroptosis.

Considering that three‐dimensional (3D) cell cultures possess more similarity to the native *in vivo* tumor state [53], we generated tumor spheroids from SiHa and C4I cells and evaluated the effects of the above ferroptosis inducer compounds on the cervical cancer cells 3D model. To determine the effective inhibitory concentrations of drugs, we tested increasing doses of each compound, starting from the highest concentration used for the 2D models of BSO and SAS, and from 5 μm for ER and from 2.5 μm for RSL3 (Fig. 1B). Spheroid size and integrity were visualized using phase contrast microscopy. The results showed that none of the drugs tested were able to inhibit the growth of SiHa and C4I 3D spheroids after 96 h of exposure to the various doses of the indicated compound (Fig. 1B).

### 3.2 DMF inhibits proliferation and promotes cell death of cervical cancer cells

**Fig. 2:**
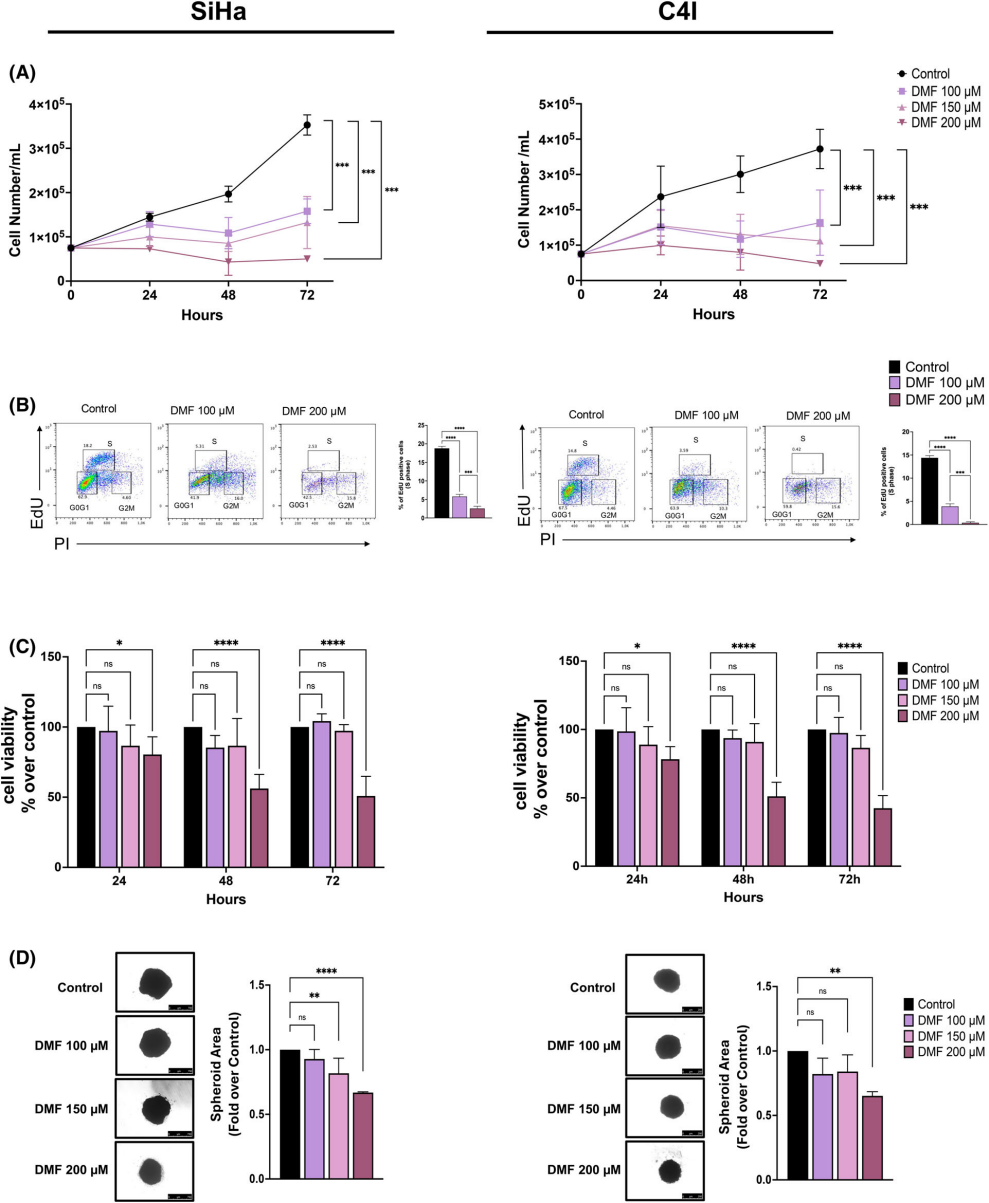
Dimethyl fumarate (DMF) reduces the growth and the viability of cervical cancer cells in 2D and 3D models. (A) 0.75 × 10^5^ SiHa and C4I cells were plated and cultured overnight before starting treatments with increasing doses of DMF (100, 150, 200 μm) for 24, 48, and 72 h or with vehicle alone (control). Control and treated cells were trypsinized and counted at indicated time to evaluate the growth. Data are expressed as cell number·mL^−1^ and presented as mean ± SD from independent experiments (*n* = 3). Statistical significance was assessed by two‐way ANOVA: ***P ≤ 0.001. (B) SiHa and C4I cells were treated with DMF (100 or 200 μm) or vehicle control for 48 h. 5‐ethynyl‐2′‐deoxyuridine (EdU) incorporation was used to quantify actively proliferating cells (S phase), and propidium iodide (PI) staining was used to assess total DNA content and cell cycle distribution. Data are shown as representative dot plots (left) and histograms of EdU‐positive cells (%) with mean ± SD (right). Statistical significance was determined by one‐way ANOVA: ***P ≤ 0.001, ****P ≤ 0.0001 (*n* = 3). (C) SiHa and C4I cell viability was assessed upon exposure of cells to vehicle (control) or to DMF at 100, 150, and 200 μm for 24, 48, and 72 h by using CCK‐8 assay. Results are reported as relative percentages of optical density obtained in treated cells compared to cells treated with vehicle (control). Data are presented as mean ± SD from independent experiments (*n* = 4). Statistical significance was assessed by two‐way ANOVA: *P ≤ 0.05; ****P ≤ 0.0001; ns, not statistically significant. (D) 3D spheroids from SiHa and C4I cells (see Section 2) were exposed for 96 h to vehicle (control) or various doses of DMF (100, 150, 200 μm). Representative images of 3D cultures were captured by inverted microscopy and quantification of spheroid area was performed as described in Section 2. All the values are reported as the mean relative area from three independent experiments, each performed in triplicate, setting as 1 the values obtained in the spheroids treated with vehicle. Data are presented as mean ± SD from independent experiments (*n* = 3). Statistical significance was assessed by one‐way ANOVA: **P ≤ 0.01; ****P ≤ 0.0001; ns, not statistically significant (Scale bar: SiHa, 750 μm or C4I, 250 μm).

DMF exhibits cytotoxic effects towards several kinds of cancer cells [44, 45, 46] and demonstrates antitumor activity by reducing tumor growth and metastasis [45, 54, 55, 56]. Therefore, we assessed the inhibitory effect of DMF on the growth of SiHa and C4I cells. As shown in Fig. 2A, single‐dose administrations of DMF at various concentrations (100, 150, and 200 μm) inhibited cell proliferation in a time‐ and dose‐dependent manner, compared with the dimethyl sulfoxide (DMSO) control group. To further confirm the inhibitory effect of DMF on cell proliferation, EdU incorporation assays were performed following treatments with DMF at 100 and 200 μm for 48 h. As shown in Fig. 2B, the proportion of actively proliferating cells, as determined by positive EdU staining, was significantly reduced in a dose‐dependent manner in both SiHa and C4I cells. Specifically, DMF 100 μm reduced cells undergoing S phase by 3.2‐fold in SiHa and 3.7‐fold in C4I, while DMF at a doubled dose of 200 μm impaired S phase by 7.3‐fold in SiHa and 39‐fold in C4I, compared to vehicle‐treated cells. These EdU staining results were consistent with the reduction in cell numbers observed in the cell‐counting assay (Fig. 2A). Notably, the inhibition of proliferation was accompanied by a marked decrease in cell number following DMF treatment, suggesting the induction of cell death. Supporting this observation, cell viability analysis using the CCK‐8 assay revealed that DMF at 200 μm exerted a pronounced cytotoxic effect in a time‐dependent manner relative to vehicle‐treated cells (Fig. 2C).

As control of DMF treatments, we analyzed the STAT3 survival signaling pathway, which is known to be crucial for cervical cancer cell survival [57]. Consistent with previous studies [55, 56, 58] we observed a marked decrease in STAT3 phosphorylation, demonstrating that DMF negatively affects STAT3‐mediated survival signaling (Fig. S2).

To evaluate whether DMF can suppress the growth of multicellular tumor spheroids derived from SiHa and C4I cells, we treated 3D cultures with single doses of 100, 150, and 200 μm DMF for 4 days. Data analysis revealed that spheroids treated with DMF (200 μm) showed a significant reduction in size, consisting of approximately 30% reduction compared to the DMSO‐treated control group (Fig. 2D). These findings confirm that DMF at 200 μm is effective in inhibiting growth in 3D cervical cancer cell models.

### 3.3 DMF at 200 μm induces ferroptosis in cervical cancer cells

**Fig. 3:**
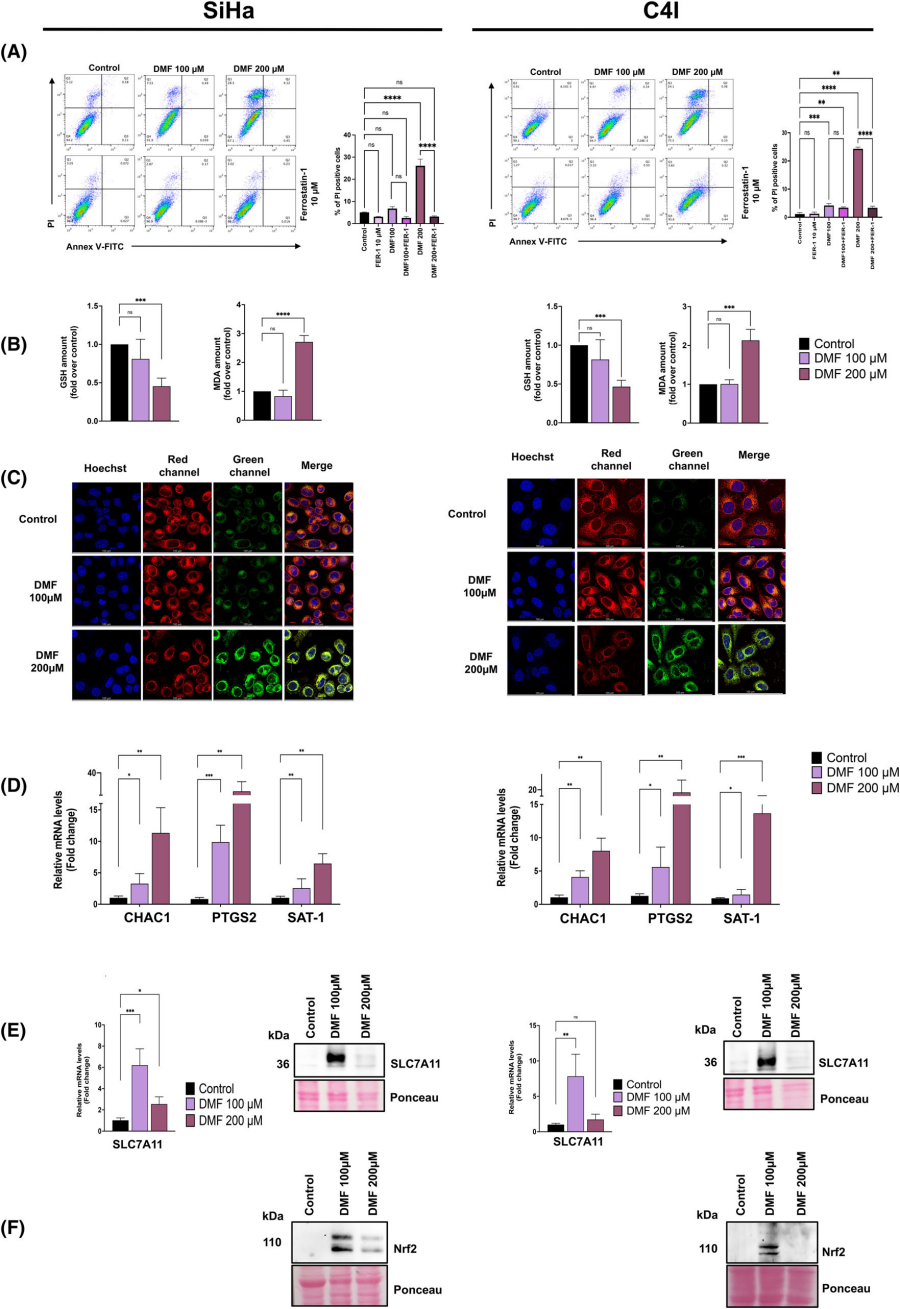
Treatment with dimethyl fumarate (DMF) at 200 μm induces ferroptosis in cervical cancer cell lines. (A) SiHa and C4I cells were treated with indicated doses of DMF in presence or absence of ferrostatin (FER‐1) at 10 μm for 48 h. Cell death was assessed by Annexin V/propidium iodide (PI) staining and flow cytometry that are shown as representative dot plots (left) and quantification of PI‐positive cells (%) presented as histograms with mean ± SD (right). Statistical significance was determined by one‐way ANOVA: **P ≤ 0.01; ***P < 0.001; ****P ≤ 0.0001; ns, not statistically significant (*n* = 3). (B) Glutathione (GSH) (left panels) and malondialdehyde (MDA) (right panels) changes in SiHa and C4I cells upon exposure to DMF (100 or 200 μm) or vehicle (control) for 6 h, were measured as described in Section 2. The data are expressed as a change relative to control that was set equal to 1 and presented as mean ± SD from independent experiments (*n* = 3). Statistical significance was assessed by one‐way ANOVA: ***P ≤ 0.001; ****P ≤ 0.0001; ns, not statistically significant. (C) Live lipid peroxidation assays on SiHa and C4I cells exposed to DMF (100 or 200 μm) or to vehicle (control) for 6 h. Representative images of fluorescence stained using the BODIPY* 581/591 C11 probe for lipid peroxides, and the Hoechst 33342 for nuclei (see Section 2). Maximum projection of Z slices is shown. Scale bar: 100 μm. (D) Expression of ferroptosis‐linked CHAC1, PTGS2, SAT‐1 genes in SiHa and C4I cells exposed to DMF (100 or 200 μm) or vehicle control for 14 h. Relative mRNA changes were assessed by using RT‐qPCR, as described in Section 2. Data are presented as mean ± SD from independent experiments (*n* = 3). Statistical significance was calculated using Student *t*‐test. *P < 0.05; **P < 0.01; ***P < 0.001. (E) mRNA and protein levels of SLC7A11 in SiHa and C4I cells treated with DMF (100 or 200 μm) or vehicle control for 24 h. Relative mRNA changes of SLC7A11 were assessed by RT‐qPCR, as described in Section 2. Data are presented as mean ± SD from independent experiments (*n* = 3). Statistical significance was calculated using Student *t* test. *P < 0.05; **P < 0.01; ***P < 0.001; ns, not statistically significant. SLC7A11 protein levels were assessed by western blotting, Ponceau S was used for loading control (*n* = 3). (F) Protein levels of Nrf2 were assessed by western blotting in SiHa and C4I cells treated with 100 or 200 μm DMF or vehicle control for 24 h (*n* = 3).

DMF has previously been shown to induce ferroptosis in diffuse large B‐cell lymphoma (DLBCL) [58]. Based on these results, we investigated whether treatment with DMF (200 μm) can activate this type of cell death in cervical cancer. To this end, SiHa and C4I cells were treated with DMF at 100 and 200 μm for 48 h, and DMF‐induced cell death was assessed by Annexin‐V/PI assay in flow cytometry. Results of Fig. 3A clearly show a dose‐dependent PI incorporation upon DMF treatment, with a statistically significant effect at 200 μm. Interestingly, annexin‐V binding was not observed, suggesting that DMF‐induced cell death does not resemble apoptosis. The co‐treatment with 10 μm Ferrostatin‐1 (FER‐1), a well‐known ferroptosis inhibitor, significantly rescued cell viability in both cell lines upon DMF treatments, at 48 h (Fig. 3A) and at 72 h (Fig. S3A), hence suggesting that DMF‐induced cell death likely occurs through the mechanism of ferroptosis. The effect of Ferrostatin‐1 on the cell viability improvement was also observed by CCK‐8 assays (Fig. S3B). Such results indicate that the dose of DMF at 200 μm induces ferroptosis in cervical cancer cells.

Given that ferroptosis is typically associated with depletion of GSH and increase of malondialdehyde (MDA), a product of polyunsaturated fatty acid peroxidation, we measured the GSH and MDA levels upon treatments with DMF (100 and 200 μm for 6 h) in both cell lines. The results indicate that GSH level strongly declines in SiHa and C4I cells treated with DMF at 200 μm and this correlates with a significant increase of MDA in both cell lines (Fig. 3B), whereas their levels were not affected by DMF at 100 μm. It is well established that GSH depletion inhibited GPX4, a key enzyme catalyzing the conversion of phospholipid hydroperoxides into corresponding phospholipid alcohols [35, 36], consequently favoring accumulation of lipid peroxides. Therefore, the Image‐iT Lipid Peroxidation Sensor BODY* 581/591 C11 probe was used to test if DMF provoked lipid peroxides in live cells. The results reported in Fig. 3C showed that in SiHa cells treated with vehicle fluorescence signals are present in the red channel that shift to green upon DMF treatments at 200 μm for 6 h, hence suggesting presence of lipid peroxides. Similar results were obtained in C4I cells, albeit 100 μm DMF treatment provokes a slight increase of green signal that was not accompanied by subsequent death (see Fig. 2B). These results confirmed that DMF at 200 μm can induce lipid peroxidation in SiHa and C4I cells following GSH depletion.

To confirm ferroptosis pathway activation at the molecular levels, we evaluated the expression of typical ferroptosis‐associated genes like glutathione‐specific gamma‐glutamylcyclotransferase 1 (CHAC1), prostaglandin‐endoperoxide synthase 2 (PTGS2), and spermidine/spermine N1‐acetyltransferase 1 (SAT1) by assessing the relative mRNA levels at 14 h post‐DMF treatments. The results reported in Fig. 3D demonstrate that 200 μm DMF strongly induces CHAC1, PTGS2, and SAT1 transcripts at in SiHa and C4I cells. The DMF dose of 100 μm also induced significant increases of these mRNAs in both cell lines; however, the downstream outcomes of DMF at 100 and 200 μm are markedly different, as shown in the CCK‐8 experiments (see Fig. 2C). This suggests that HPV‐positive cervical cells under DMF at 100 μm retain the ability to activate molecular pathways counteracting ferroptosis, whereas at 200 μm these protective mechanisms are impaired. Thus, DMF at 100 μm elicits an adaptive antioxidant response that limits the execution of ferroptosis, whereas at 200 μm this protective program fails, resulting in GSH collapse, lipid peroxidation, and ferroptotic cell death.

Since it is known that DMF reacts with and depletes GSH [46] and that cells can subsequently recover GSH via *de novo* synthesis mainly through SLC7A11‐mediated cystine import [19, 38, 59, 60, 61], we analyzed the expression levels of this gene after DMF treatments. Real‐time PCR and western blot analyses demonstrated that treatments with DMF (100 μm) of SiHa and C4I cells provoked a marked increase of SLC7A11 expression compared to that treated with vehicle control. In contrast, cells treated with DMF (200 μm) showed a slight induction of SLC7A11 mRNAs without protein increase (Fig. 3E). To better understand the molecular pathway/s mediating such transcriptional regulation, we examined the levels of Nrf2, a positive regulator of SLC7A11, under both concentrations of DMF. As shown in Fig. 3F, Nrf2 strongly accumulates under DMF (100 μm) treatments; however, its levels are poorly induced/not increased upon treatments with 200 μm DMF.

These molecular findings indicate that, in cervical cancer cells, DMF at 100 μm is associated with activation of the Nrf2 pathway and induction of SLC7A11 transcription.

Consistent with increased SLC7A11 expression, cells exposed to 100 μm DMF efficiently maintain intracellular GSH levels, thereby preventing execution of ferroptosis. In contrast, treatment with DMF at 200 μm results in weak Nrf2‐dependent induction of SLC7A11 mRNA and absence of the corresponding protein, leading to impaired cystine import, progressive GSH depletion, and ferroptotic cell death in 2D cultures, together with inhibition of 3D tumor growth (Fig. 2C).

### 3.4 Subcytotoxic concentrations of DMF enhance the sensitivity of cervical cancer cells to low doses of cisplatin

Cisplatin (CDDP) represents the most common drug used for cervical cancer treatment [62]. CDDP reacts with DNA forming adducts that inhibit DNA replication, hence fostering apoptosis. Despite its potency, cisplatin treatments are associated with adverse effects including nephro‐ and oto‐toxicity, as well as resistance development, hence limiting its clinical use. To achieve a better clinical outcome, cisplatin often is combined with other therapies [63]. For this reason, we explored whether the co‐administration of DMF plus cisplatin, both used at subcytotoxic doses, shows effects on viability of cervical cancer cell models.

We evaluated CDDP cytotoxicity at escalating doses (20, 40, and 80 μm) using CCK‐8 assays and determined that the IC_50_ values for SiHa and C4I cells are approximately 40 μm following 72 h of treatment (Fig. S4A). Western blot analysis at 24 h revealed cleavage of PARP‐1, a DNA repair enzyme, in both cell lines treated with 80 μm CDDP (Fig. S4B). In addition, we observed a dose‐dependent increase in caspase 3/7 activity, at 24 h, starting at 40 μm CDDP in C4I cells (Fig. S4B). Overall, the data indicate that in cervical squamous cell carcinoma cells, CDDP promotes the induction of apoptotic pathways, as reported before for adenocarcinoma HeLa cervical cells [64].

**Fig. 4:**
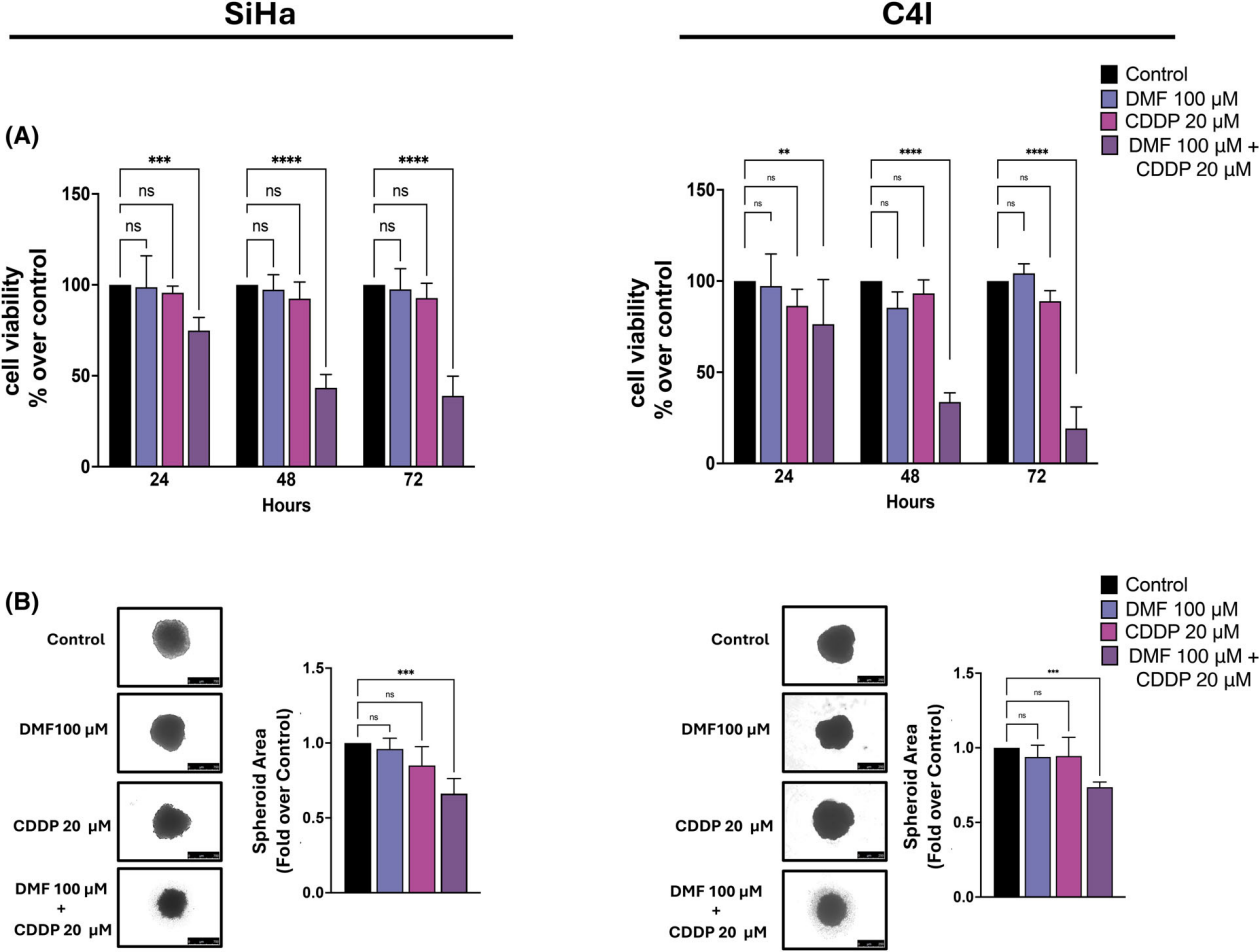
Treatment with dimethyl fumarate (DMF) at 100 μm significantly increases the susceptibility of cervical cancer cell models to low‐dose cisplatin. (A) SiHa and C4I cells were exposed to vehicle (control), DMF (100 μm) or Cisplatin (CDDP, 20 μm) as well as the combination of both drugs for various times (24, 48, or 72 h). Cell viability was determined by using CCK‐8 assay. Data are expressed as relative percentages of optical density obtained in treated cells compared to control. Data represent means ± SD from independent experiments (*n* = 5). Statistical significance was assessed by two‐way ANOVA: **P ≤ 0.01; ***P ≤ 0.001; ****P ≤ 0.0001; ns, not statistically significant. (B) 3D spheroids derived from SiHa and C4I cells were treated for 96 h with vehicle (control) or DMF (100 μm) or CDDP (20 μm) as well as with DMF plus CDDP. Representative images of 3D cultures were captured by inverted microscopy and quantification of spheroid area was performed as described in Section 2. All the values are reported as the mean relative area from three independent experiments, each performed in triplicate, setting as 1 the values obtained in the spheroids treated with vehicle (control). Data represent means ± SD from independent experiments (*n* = 3). Statistical significance was assessed by one‐way ANOVA: ***P ≤ 0.001; ns, not statistically significant (Scale bar: SiHa, 750 μm or C4I, 250 μm).

Given that DMF sensitizes cervical cancer cells to ferroptosis and CDDP to apoptosis, we aimed to evaluate whether the combination of these drugs acting with different cell death mechanisms improves the vulnerability of cervical cancer cells. With the aim to test combination of drugs at subcytotoxic concentrations, we chose CDDP 20 μm (below the IC_50_), and DMF at 100 μm unaffecting cell viability for the cotreatment experiments. SiHa and C4I cells were treated with vehicle, DMF, or CDDP alone or with both drugs, and their impact on cell viability was examined through CCK‐8 assays. As demonstrated in Fig. 4A, both cell lines exhibit a strong significant reduction in viability under combination of DMF (100 μm) with 20 μm CDDP in a time‐dependent manner, whereas single drugs have no effects, as expected by the above results.

Furthermore, we assessed if this combination produces similar effects also in cervical cancer 3D models. Results shown in Fig. 4B indicate that a single co‐administration for 96 h of DMF (100 μm) and CDDP (20 μm) significantly reduced the size of SiHa and C4I spheroids compared to the control groups, indicating that also in more physiologically relevant cell models the combination of these drugs renders cells less prone to growth.

### 3.5 Combination of subcytotoxic doses of DMF and cisplatin drives death pathways of ferroptosis and/or apoptosis

**Fig. 5:**
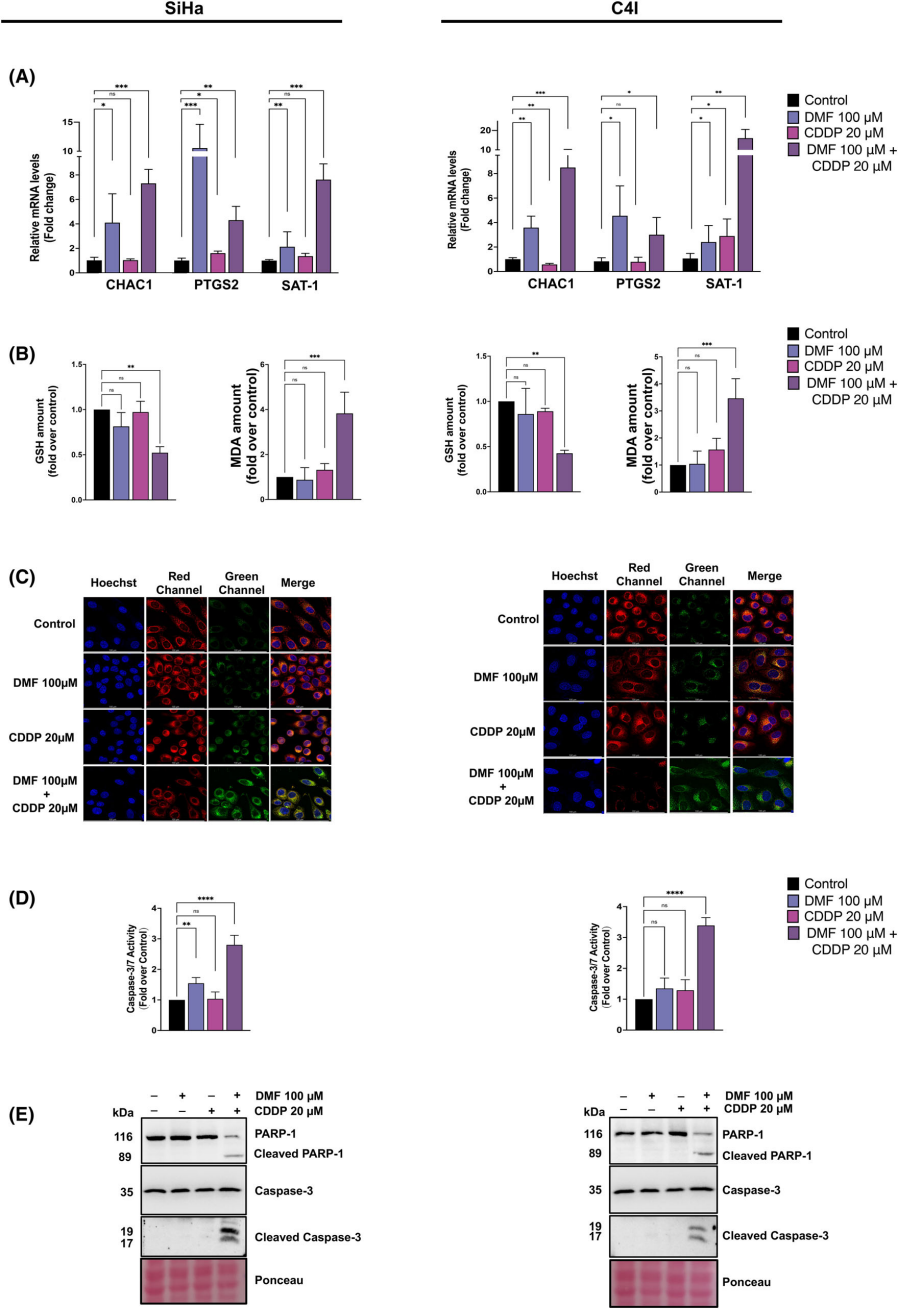
Cervical cancer cells treated with dimethyl fumarate (DMF) plus cisplatin (CDDP) undergo both ferroptosis and apoptosis. (A) SiHa and C4I cells were exposed to the indicated concentrations of DMF, CDDP, or DMF plus CDDP as well as to vehicle (control) for 14 h. Relative mRNA changes of ferroptosis‐linked CHAC1, PTGS2, SAT‐1 genes were evaluated by using RT‐qPCR, as described in Section 2. Data represent means ± SD from independent experiments (*n* = 3). Statistical significance was calculated using Student *t*‐test. *P < 0.05; **P < 0.01; ***P < 0.001; ns, not statistically significant. (B) Glutathione (GSH) (left panels) and malondialdehyde (MDA) (right panels) changes in SiHa and C4I cells treated with the indicated concentrations of DMF or CDDP or DMF plus CDDP as well as with vehicle (control) for 6 h, were measured as described in Section 2. The data are expressed as a change relative to control that was set equal to 1. Data represent means ± SD from independent experiments (*n* = 3). Statistical significance was assessed by one‐way ANOVA: **P < 0.01; ***P < 0.001; ns, not statistically significant. (C) Live lipid peroxidation assays on SiHa and C4I cells exposed to DMF, CDDP or DMF plus CDDP as well as with vehicle (control) for 6 h. Representative fluorescence images stained using the BODIPY* 581/591 C11 probe for lipid peroxides, and the Hoechst 33342 for nuclei (see Section 2). Maximum projection of Z slices is shown. Scale bar: 100 μm. (D) Caspase 3/7 activities in SiHa and C4I cells exposed to DMF or CDDP or DMF plus CDDP as well as to vehicle for 24 h were assessed as described in Section 2. The data are expressed as a change relative to control that was set equal to 1. Data represent means ± SD from independent experiments (*n* = 3). Statistical significance was assessed by one‐way ANOVA: **P ≤ 0.01; ****P ≤ 0.0001; ns, not statistically significant. (E) Western blot analysis of PARP1 and caspase‐3 cleavage was performed upon exposure of SiHa and C4I cells to DMF or CDDP or DMF plus CDDP as well as to vehicle (−) for 24 h. Ponceau S was used for loading control (*n* = 3).

Next, we asked whether cell death observed under simultaneous administration of subcytotoxic doses of DMF (100 μm) and CDDP (20 μm) derives from activated ferroptosis and/or apoptosis. Therefore, we evaluated specific markers of both ferroptosis and apoptosis in SiHa and C4I cells. As shown in Fig. 5A, DMF alone induces mRNA levels of ferroptosis‐associated genes CHAC1, PTGS2, and SAT1 but without cell death (see also Figs 2C and 3D). DMF plus CDDP administration produces significant increases of the same mRNAs compared to control cells. Of note, while CHAC1 and SAT1 transcripts were potentiated by DMF plus CDDP, the PTGS2 mRNA was less induced with respect to DMF alone. The specific reason for this PTGS2 down‐modulation was not investigated.

It has been reported that DMF as well as CDDP can directly react with GSH [46, 65], therefore we assessed whether combination of subcytotoxic DMF and CDDP can reduce cellular GSH amount. Results of GSH assays demonstrated that in both SiHa and C4I cells, the GSH levels strongly declined upon DMF plus CDDP treatments for 6 h compared to control groups (Fig. 5B). Further analysis on MDA content and lipid peroxide production revealed a statistically significant increase in MDA (Fig. 5B) and presence of lipid peroxides (Fig. 5C) in both cell lines treated with DMF plus CDDP compared to control groups. Taking together these data indicate that DMF in combination with CDDP led to strong GSH depletion and consequently to ferroptosis, as demonstrated by the presence of specific markers.

Regarding apoptosis, we found that caspase‐3/7 activity was significantly increased in cells (both types, SiHa and C4I) treated with DMF plus CDDP (Fig. 5D) compared to control. In parallel, we observed a reduction of the full‐length PARP‐1 protein, associated with an increase in its inactive cleaved form (Fig. 5E) derived from caspase‐3/7 activity during the execution phase of apoptosis, suggesting that apoptosis is a concomitant mechanism of cell death activated after DMF plus CDDP treatments.

### 3.6 Cell death in cervical cancer induced by the combination of subcytotoxic DMF and cisplatin involves p53‐mediated suppression of SLC7A11

**Fig. 6:**
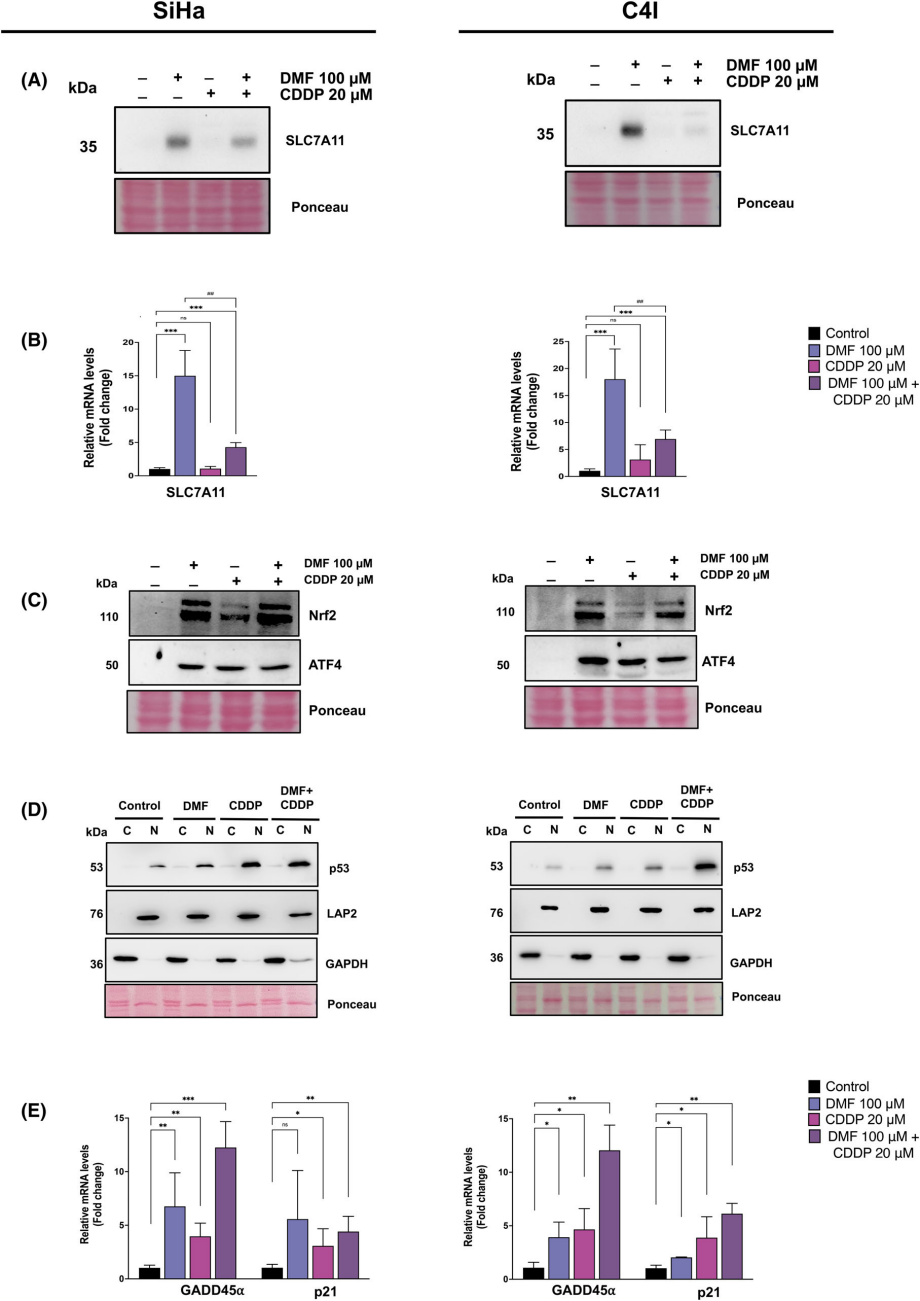
p53‐mediated suppression of SLC7A11 is implicated in cervical cancer cell death induced by subcytotoxic doses of dimethyl fumarate (DMF) plus cisplatin (CDDP). (A) SiHa and C4I cells were exposed to vehicle (−) or to DMF (100 μm), CDDP (20 μm) or combined drugs. Western blot analyses of SLC7A11 protein were performed at 24 h upon treatments. Ponceau S was used for loading control (*n* = 3). (B) RT‐qPCR analysis was employed to measure mRNA levels of SLC7A11 in SiHa and C4I cells treated for 14 h with vehicle (control) and with the indicated drugs. Data are presented as mean ± SD from independent experiments (*n* = 3). Statistical significance was calculated using Student *t*‐test. ***P < 0.001; ^##^ *P* < 0.01; ns, not statistically significant. (C) Western blot analyses of Nrf2 and ATF4 were performed upon exposure of SiHa and C4I cells to DMF or CDDP or DMF plus CDDP as well as to vehicle (−) for 24 h. Ponceau S was used for loading control (*n* = 3). (D) Cytosolic (C) and nuclear (N) p53 levels were assessed by western blot analyses after 24 h of treatments with vehicle (control), or DMF (100 μm), CDDP (20 μm), or with both drugs. LAP2 and GAPDH were used as loading controls for the nuclear and cytosolic fractions, respectively. The blot is representative of three independent experiments. (E) SiHa and C4I cells were exposed to indicated drugs as well as to vehicle (control) for 14 h. Relative mRNA changes of p53‐linked GADD45α and p21 genes were evaluated by using RT‐qPCR, as described in Section 2. Data are presented as mean ± SD from independent experiments (*n* = 3). Statistical significance was calculated using Student *t*‐test. *P < 0.05; **P < 0.01; ***P < 0.001; ns, not statistically significant.

SLC7A11 activity is necessary to preserve intracellular GSH levels [19, 38]. To ascertain if the decrease in GSH amount revealed upon noncytotoxic DMF plus CDDP treatments (Fig. 5B) could be related to the GSH biosynthesis process, we analyzed SLC7A11 expression using western blotting in SiHa and C4I cells treated with DMF (100 μm), CDDP (20 μm) or both for 24 h (Fig. 6A). As expected, the results demonstrated that DMF alone induces the expression of SLC7A11 in cervical cancer cells compared to that treated with vehicle control (see also Fig. 3E); however, in both cell types exposed to combination of DMF and CDDP, we revealed a remarkable reduction of SLC7A11 protein levels, compared to those observed in cells treated with DMF alone (Fig. 6A). To investigate a possible transcriptional modulation in SLC7A11 expression pattern, we measured the mRNA levels of SLC7A11 across treatments (Fig. 6B). The results demonstrate that SLC7A11 transcripts significantly decreased in cells treated with DMF plus CDDP compared with the control groups and with DMF‐treated groups (Fig. 6B).

As Nrf2 and ATF4 are known positive regulators of SLC7A11 transcription, we investigated their involvement under these conditions. Results showed that in both SiHa and C4I cells, Nrf2 and ATF4 protein levels are also increased upon treatments with DMF (100 μm) alone compared with control groups and remain elevated in DMF plus CDDP (Fig. 6C). Of note, CDDP alone modestly increases Nrf2 and induces ATF4. Remarkably, these uncoupling between sustained Nrf2/ATF4 accumulation and SLC7A11 repression represents a distinctive feature of the combined DMF and CDDP treatments.

To shed light on the molecular basis underlying the reduced SLC7A11 transcription observed in DMF plus CDDP treatments, we investigated the levels of p53 protein, considering that SLC7A11 is a direct target gene suppressed by p53 [66, 67] and cervical cancer cells possess wild‐type p53 [68]. We checked nuclear p53 accumulation by performing western blot analysis on nuclear and cytosolic protein extracts from treated cells. As demonstrated in Fig. 6D, in both SiHa and C4I cervical cells, nuclear p53 levels were mildly increased in single treatments, but they strongly elevated after DMF plus CDDP administration, compared with control cells. We hence tested the mRNA levels of GADD45α and p21, two p53‐dependent genes, and results demonstrated that both mRNAs significantly accumulate over the treatments at different degrees, with a stronger induction in the combination treatments of DMF plus CDDP confirming p53 activation (Fig. 6E).

Together, these data suggest that under DMF plus CDDP, p53 activation acts as a dominant brake on SLC7A11 expression, overreading sustained Nrf2/ATF4 accumulation and thereby preventing the cysteine‐driven GSH replacement program.

### 3.7 GSH depletion and p53 (re)activation contribute to cervical cancer cell death induced by CDDP and DMF combined treatments

The obtained data suggest that under cotreatments cell death can be activated by the coexistence of two interrelated mechanisms: GSH depletion and p53 (re)activation.

Sensitivity to ferroptosis in cervical cancer is modulated throughout the various stages of cellular transformation by increasing GCLM and GPX4 expression [34], suggesting GSH‐dependent protective mechanisms. One of these mechanisms may be mediated by SLC7A11, that primarily regulates cellular redox status, thereby inhibiting ferroptosis signaling [19, 38, 69].

**Fig. 7:**
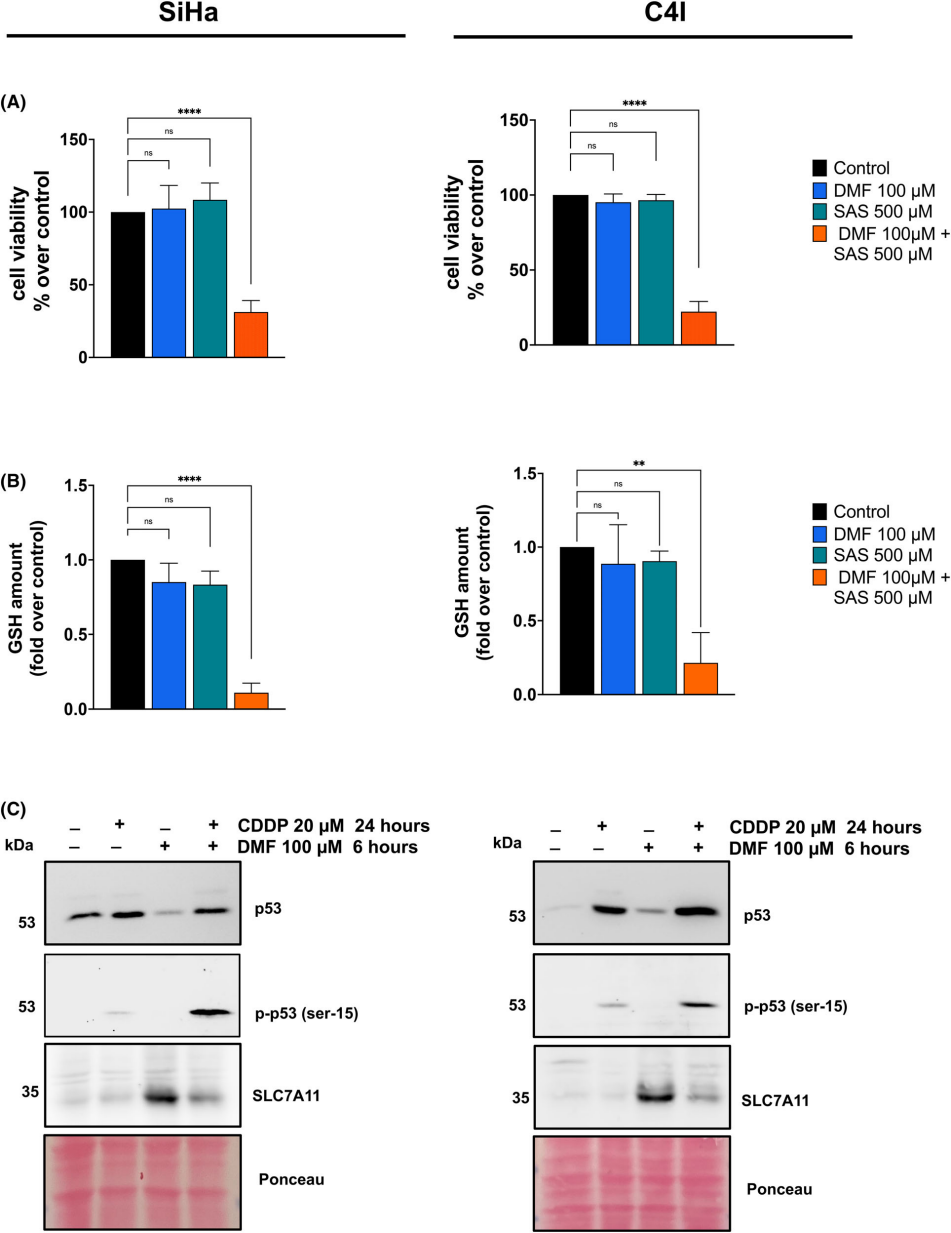
Glutathione (GSH) depletion and p53 (re)activation jointly contribute to cervical cancer cell death induced by Dimethyl fumarate (DMF) in combination with cisplatin (CDDP). (A) SiHa and C4I cells were exposed to vehicle (control) or to DMF at 100 μm, sulfasalazine (SAS) at 500 μm as well as the combination of both drugs for 48 h. Cell viability was assessed by using CCK‐8 assay. Results are expressed as relative percentages of optical density obtained in treated cells compared to control. Data are presented as mean ± SD from independent experiments (*n* = 5). Statistical significance was assessed by one‐way ANOVA: ****P ≤ 0.0001; ns, not statistically significant. (B) GSH changes in SiHa and C4I cells exposed to vehicle (control) or DMF (100 μm), SAS (500 μm) or both for 6 h, were measured as described in Section 2. The data are expressed as a change relative to control that was set equal to 1. Data are presented as mean ± SD from independent experiments (*n* = 3). Statistical significance was assessed by one‐way ANOVA: **P ≤ 0.01; ****P ≤ 0.0001 (*n* = 3); ns, not statistically significant. (C) Western blot analysis of p53, p‐p53 (ser‐15) and SLC7A11 was performed upon exposure to vehicle (−), CDDP (20 μm) for 24 h and then treated or not with DMF (100 μm) for 6 h. Ponceau S was used for loading control (*n* = 3).

Therefore, we investigated whether DMF at 100 μm can become cytotoxic in cervical cancer cells by specifically targeting SLC7A11‐mediated activity. Sulfasalazine (SAS) is a drug belonging to the sulfonamide class, composed of 5‐aminosalicylic acid and sulfapyridine [70], which is used to treat inflammatory arthritis and inflammatory bowel disease. In cancer cells, SAS can produce cytotoxic effects by inhibiting the cystine/glutamate antiporter activity [71] and has been repurposed as an anticancer drug [72, 73]. Our experiments demonstrated that in cervical cancer cells, SAS alone used at different concentrations (up to 500 μm) has no cytotoxic effects (Fig. 1). Therefore, we cotreated SiHa and C4I cells with sulfasalazine at 500 μm plus DMF at 100 μm for 48 h and measured cell viability by CCK‐8 assays. Results showed a significant marked decrease in cell viability, compared to vehicle (Fig. 7A). Moreover, because of SLC7A11 inactivation, the GSH levels were strongly reduced in DMF plus SAS treated cells compared to the control (Fig. 7B). Overall, these results demonstrate that in cervical cancer cells, the inhibition of SLC7A11 activity by SAS cannot replenish GSH, implicating an essential role of the cystine import on the cervical cancer cell viability.

It has been demonstrated that phosphorylation at Ser15 of p53 (p‐p53 Ser15) is needed to recruit more p53 on promoters after stimuli [74, 75]. Since CDDP provokes phosphorylation at Ser15 of p53 [76, 77] that can also be important for downregulation of SLC7A11, as demonstrated in other contexts [78, 79], we also analyzed the contribution of this modification. To this aim, SiHa and C4I cells were first treated with 20 μm CDDP for 24 h to induce Ser15 phosphorylation and subsequently treated with DMF (100 μm for 6 h) to increase SLC7A11 transcription. As shown in Fig. 7C, CDDP alone generates a mild p‐p53Ser15, whereas it was absent in DMF‐treated cells, a condition in which SLC7A11 protein remarkably increases. Notably, in both cell lines pretreated with CDDP and then exposed to DMF, p‐p53 Ser15 strongly increased, and this coincides with a pronounced reduction of SLC7A11 level. Therefore, DMF plus CDDP act synergistically on p‐p53Ser15 that can mediate suppression of SLC7A11. By repressing SLC7A11, p53 can provoke cysteine/GSH depletion, a condition that further exacerbates pro‐oxidant environment and DNA damage leading to apoptosis, concurrently p53 by activating SAT‐1 (Fig. 5A) reinforces ferroptosis.

In conclusion, taken together, our results suggest that SLC7A11 inhibition by combined CDDP and DMF treatments increases vulnerability of cervical cancer cells to ferroptosis and apoptosis mediated by p53 (re)activation.

## 4 Discussion

Ferroptosis arises from the accumulation of lipid peroxides, primarily fostered by GSH depletion. In fact, GSH, being an important cofactor of the GPX4 enzyme that repairs lipid peroxides into alcohols, acts as a crucial modulator of ferroptosis [35, 50]. Nevertheless, reduction in GSH levels may also lead to other forms of cell death, including apoptosis and/or autophagy [80]. For the synthesis of GSH, cells predominantly use cysteine derived from cystine (the precursor of cysteine), imported into cells through the membrane cystine/glutamate antiporter system xCT [81], that consists of two subunits, SLC7A11 and SLC3A2. Among these, SLC7A11 functions as a transporter and exchanges glutamate with cystine whereas SLC3A2 controls the stability of xCT. SLC7A11 transcription can be induced by the transcription factors Nrf2 and activating transcription factor 4 (ATF4) that in some conditions act synergistically [82, 83]. Increased SLC7A11 expression supports GSH synthesis, and this can lead to the resistance of tumor cells to ferroptosis and can also promote tumor development [69]. Interestingly, SLC7A11 can be negatively regulated by some transcription factors including p53 [67] and activating transcription factor 3 (ATF3) [84]. Therefore, the absence or inactivation of p53 renders cells more resistant to ferroptosis [66]. In some contexts, phosphorylation at Ser15 of p53 leads to downregulation of SLC7A11 transcription and upregulation of SAT1 [78, 79].

Concerning HPV‐positive cervical carcinoma cells, ferroptosis was found dysregulated during the various stages of transition from normal to cancerous squamous cells [34]. In fact, during the development of SIL, positive cells can adopt ferroptosis as a tumor suppressor mechanism. However, during transformation its persistence in some cells causes activation of ferroptosis defense mechanisms fostered by enhanced RAS signaling thereby promoting transformation towards carcinoma. Of interest, the authors found an increase of antioxidant GCLM and GPX4 under ferroptosis‐inducing conditions in cervical cancer cells, and these correlate with *in vivo* studies on tissue sections from patients at different transformation [34]. Since GCLM is an enzyme crucial for GSH biosynthesis and GPX4 activities are dependent on GSH acting as cofactor of GPX4, these results also suggest that resistance mechanisms against ferroptosis require a continuous supply of GSH. Additionally, the GSH‐mediated detoxification process is a well‐established mechanism of drug resistance, and innovative approaches that limit GSH synthesis are being researched for HPV‐related cancer cells [85]. Accordingly, previous metabolomic data by Pappa group demonstrated enrichment in cysteine and GSH amount in cervical cancer cells [37]. Furthermore, glutathione reductase, an enzyme recovering GSH, was found increased in CC tissues, and its inhibition disrupts redox homeostasis leading to an increase of ROS that elicits cell death [86]. Given that there is a dynamic modulation of susceptibility to ferroptosis in HPV‐related cancers [31] and that HPV infection can induce a state of chronic oxidative stress by increasing ROS production [87], it could be theoretically possible to activate ferroptosis in CC. In fact, ferroptosis depends on oxidative stress and although HPV‐positive cervical cancer cells cope with redox stress to counteract cell death, they can become more vulnerable to the oxidative stress milieu itself, rendering HPV‐positive cervical cancer cells vulnerable to redox‐sensitive treatments [88].

Our study revealed that DMF at 200 μm induces ferroptosis in two human cervical carcinoma cell lines and arrests growth in 3D models, while it is not cytotoxic when used at a concentration of 100 μm. It is known that DMF can exert multiple effects [89, 90, 91] on various tissues and/or systems through different mechanisms likely caused by post‐translational modifications, mainly succination on cysteine/s of various targets [92]. One of the primary recognized targets of DMF is GSH [92, 93] along with other molecules including Keap1, the negative regulator of Nrf2 transcription factor [94, 95, 96], DJ‐1/parkin7, a stabilizer of Nrf2 [97], and metabolic enzymes like GAPDH [98] and MTHFD1 [99].

Cysteine modifications of Keap1 abolish its interaction with Nrf2, leading to accumulation of Nrf2 and subsequent Nrf2‐dependent cytoprotective and antioxidant gene expression [100]. However, Saidu et al. [97] demonstrated that DMF can exhibit dose‐dependent effects in OVCAR3 cancer cells, being protective at low doses by inducing Nrf2 and cytotoxic at high doses by depleting Nrf2 through DMF‐triggered inactivation of DJ‐1. Of note, Faleti et al. recently reported that DMF in hepatoma cells induces apoptosis by targeting the Nrf2/Bcl‐xL axis, hence provoking damage to mitochondria [56, 91, 97].

Since we found that cellular GSH amounts were unchanged at 100 μm of DMF but strongly reduced at 200 μm (see Fig. 3B), we focused on SLC7A11, a key regulator of *de novo* GSH biosynthesis [19, 38, 59, 60, 61]. Our data demonstrated that cervical cancer cells treated with 100 μm DMF could activate transcription of SLC7A11 via Nrf2 activation, consistent with previous reports describing DMF‐mediated modulation of Keap1 [94, 95, 96], leading to efficient recovery of GSH content and prevention of ferroptosis. In contrast, cells treated with DMF at 200 μm showed limited induction of SLC7A11 mRNAs without detectable protein increase and this coincides with poorly/absent Nrf2 induction. Therefore, cells cannot import cystine, resulting in GSH depletion associated with ferroptosis in 2D models and inhibiting 3D‐tumor proliferation. The impaired Nrf2‐SLC7A11 activation upon DMF (200 μm) featuring GSH depletion needs further investigation, but it nicely reflects the ability of cervical cancer to acquire/maintain sensitivity to ferroptosis by means of SLC7A11/GSH axis targeting. These results are consistent with literature data showing that in cancer cells, DMF could downregulate Nrf2 if used at cytotoxic dose [56, 91, 97].

DMF is a therapeutic drug currently used to treat patients with psoriasis or multiple sclerosis [90, 101, 102]. It has been reported that DMF can exert pleiotropic effects, depending on the dose and context used. Generally, low (specific) doses of DMF have anti‐inflammatory and immunoregulatory effects through modulation of nuclear factor kappa B and the STAT3 pathway and are cytoprotective in neurodegenerative conditions by increasing Nrf2 activity [103, 104]. High/specific doses of DMF can produce harmful effects by depleting GSH hence making cancer cells more vulnerable to oxidative damage [105] including KRAS mutated cancer cells [81], melanoma [106], colon carcinoma [107], and oral squamous cell carcinoma [108]. Hence, DMF may be a valuable drug to repropose for arresting tumor growth and metastasis (recently reviewed by Zhang et al. [109]), and we demonstrate its effectiveness in HPV‐positive cervical cancer cells and derived‐3D models.

By killing tumor cells with different mechanisms, combined therapy brings advantages over single‐drug treatments [63, 110, 111]. Specifically, under combined therapy a greater quantity of cancer cells could be targeted, the risk of resistance can be reduced, and by using lower doses of drugs, toxicity as well as side effects can be minimized [63, 112]. DMF has proven an ideal combination therapy partner of oxaliplatin in colorectal cancer [107], vemurafenib in melanoma [113], and venetoclax in acute myeloid leukemia (AML) cell lines [114].

A limitation of this study is the absence of an animal model, which prevents full evaluation of DMF's efficacy and potential side effects *in vivo*. Although our 2D and 3D models provide valuable insights into its effects on cervical cancer cells, *in vivo* experiments will be necessary to confirm these findings and assess pharmacokinetics, toxicity, and systemic responses.

Our data demonstrated that HPV‐positive cervical cancer cells become vulnerable when treated with a combination of DMF (100 μm) and cisplatin (20 μm), concentrations that are not cytotoxic when administered individually. This appears to depend on p53 levels that may promote or inhibit both ferroptosis (reviewed in Refs [115, 116]) and apoptosis (reviewed in Ref. [117]). Our experiments demonstrate that DMF (100 μm) alone induces only limited nuclear p53 accumulation, which may elicit antioxidant effects, impact positively on cell cycle arrest/DNA repair and be unable to repress SLC7A11 transcription that is activated via Nrf2 accumulation; in fact, literature data support that relatively low levels of p53 decrease oxidative stress [118]. About 20 μm CDDP alone also provokes a nuclear modest p53 induction that can favor cell cycle arrest and repair of DNA damage primed by CDDP. The experiments on combined DMF plus CDDP prove that the mechanism implicated in death involves a synergistic activation of p53 by DMF and CDDP (Fig. 6); consistently, nuclear p53 levels are more increased upon exposure to DMF plus CDDP, therefore promoting both ferroptosis and apoptosis. Under combined DMF and CDDP treatment, p53 activation acts as a dominant brake on SLC7A11 expression, overriding Nrf2/ATF4‐associated pro‐survival signaling and preventing cystine‐dependent GSH replenishment. Indeed, DNA damage‐dependent p53 activation also produces oxidative stress, essential in p53‐dependent apoptosis [119, 120]. Therefore, we propose that the combination of CDDP and DMF establishes a positive feedback loop that amplifies both oxidative stress and DNA damage. At molecular levels, activated high p53 suppresses SLC7A11 expression, therefore cells cannot import cystine to synthesize GSH. In turn, GSH depletion favors a more pro‐oxidant milieu and further DNA damage that jointly reinforce activation of p53 inducing both ferroptosis [66] and apoptosis [119, 120]. Accordingly, we found that p‐p53 Ser15 was absent in cells treated with DMF alone, mild increased in CDDP alone, but strongly enhanced in cells pretreated with CDDP and then exposed to DMF, suggesting a synergistic p53 activation by drug combination. This late event coincides with decreased SLC7A11. On this basis, we propose that DMF plus CDDP induces cell death in HPV‐positive cervical cancer cells through convergent mechanisms centered on GSH depletion triggered by p53 (re)activation/SLC7A11 axis.

Collectively, our data demonstrated that in this cancer context, GSH depletion achieved either by impaired Nrf2‐SLC7A11 activation (with DMF at 200 μm alone) or by SLC7A11 repression caused by (re)activated p53 (under combined DMF plus CDDP treatments) may have relevant therapeutic implications.

## 5 Conclusions

This work reported that GSH depletion either induced by DMF at 200 μm or by the combination of DMF plus CDDP drives cell death in cervical cancer cells and inhibits growth in spheroid models. At the molecular level, we showed that DMF (200 μm) impairs Nrf2‐SLC7A11‐dependent *de novo* GSH synthesis, therefore promoting ferroptosis in 2D models and inhibiting 3D‐tumor proliferation. DMF plus CDDP promotes p53 (re)activation that suppresses SLC7A11 expression thereby triggering both ferroptosis and apoptosis. In conclusion, we provide a molecular basis for considering DMF alone or DMF plus cisplatin administration a new avenue to be explored in cervical cancer treatment.

## Conflict of interest

The authors declare no conflict of interest.

## Author contributions

RF and MLT contributed to conceptualization; CP, GM, MLT, and RF contributed to methodology; CP, SR, LM, and RF contributed to formal analysis; CP and GM contributed to investigation; RF and MLT contributed to resources; CP and GM contributed to data curation; CP, GM, MLT, and RF contributed to writing‐original draft preparation; CP, GM, SR, KA, MLT, and RF contributed to writing‐review and editing; RF and MLT contributed to supervision; KA and MLT contributed to funding acquisition. All authors have read and agreed to the published version of the manuscript.

## Supporting information


**Fig. S1.** Effect of ferroptosis inducers on cell viability of Caski and HeLa cells.
**Fig. S2.** Effect of dimethyl fumarate (DMF) on STAT3 signaling.
**Fig. S3.** Dimethyl fumarate (DMF) at 200 μm induces ferroptosis in SiHa and C4I cells.
**Fig. S4.** Dose‐dependent effects of cisplatin (CDDP) on SiHa and C4I cells.
**Table S1.** Primers used for RT‐qPCR analyses.

## Data Availability

All data reported in this study are available in this published article and in its Supporting Information.
