# Supplementary material for: Dimethyl fumarate combined with cisplatin at subcytotoxic doses sensitizes cervical cancer toward ferroptosis and apoptosis through GSH restriction and p53 (re)activation
Source: Mol Oncol. 2026 Mar 25:10.1002/1878-0261.70216. Online ahead of print. doi: 10.1002/1878-0261.70216 (PMC13398358; doi:10.1002/1878-0261.70216)
Supplement: Supplementary file 1 — Fig. S1. Effect of ferroptosis inducers on cell viability of Caski and HeLa cells. Fig. S2. Effect of dimethyl fumarate (DMF) on STAT3 signaling. Fig. S3. Dimethyl fumarate (DMF) at 200 μm induces ferroptosis in SiHa and C4I cells. Fig. S4. Dose‐dependent effects of cisplatin (CDDP) on SiHa and C4I cells. Table S1. Primers used for RT‐qPCR analyses. [file MOL2-9999-0-s001.pdf]

Figure S1

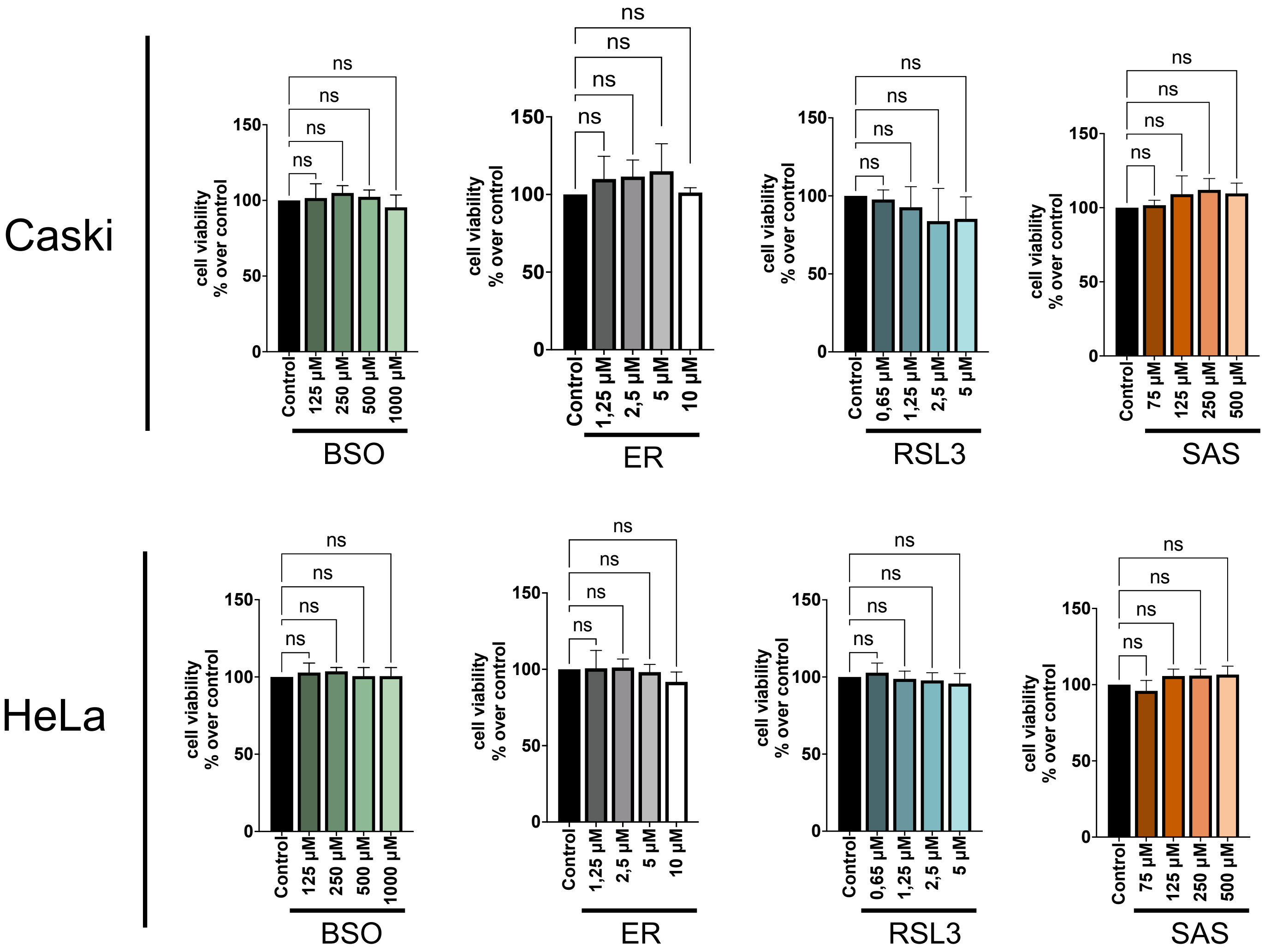

Figure S1. Effect of ferroptosis inducers on cell viability of Caski and HeLa cells. Caski and HeLa cells were treated with the indicated concentrations of drugs [Buthionine Sulfoximine (BSO), Erastin (ER), RAS-selective lethal 3 (RSL3), or Sulfasalazine (SAS)] for 24 hours. Cell viability was assessed by CCK-8 assay. Data are reported as relative percentages of optical density obtained in treated cells compared to cells treated with vehicle (control). Data are presented as mean ± SD from independent experiments (n = 4). Statistical significance was assessed by one-way ANOVA: ns = not statistically significant.

Figure S2

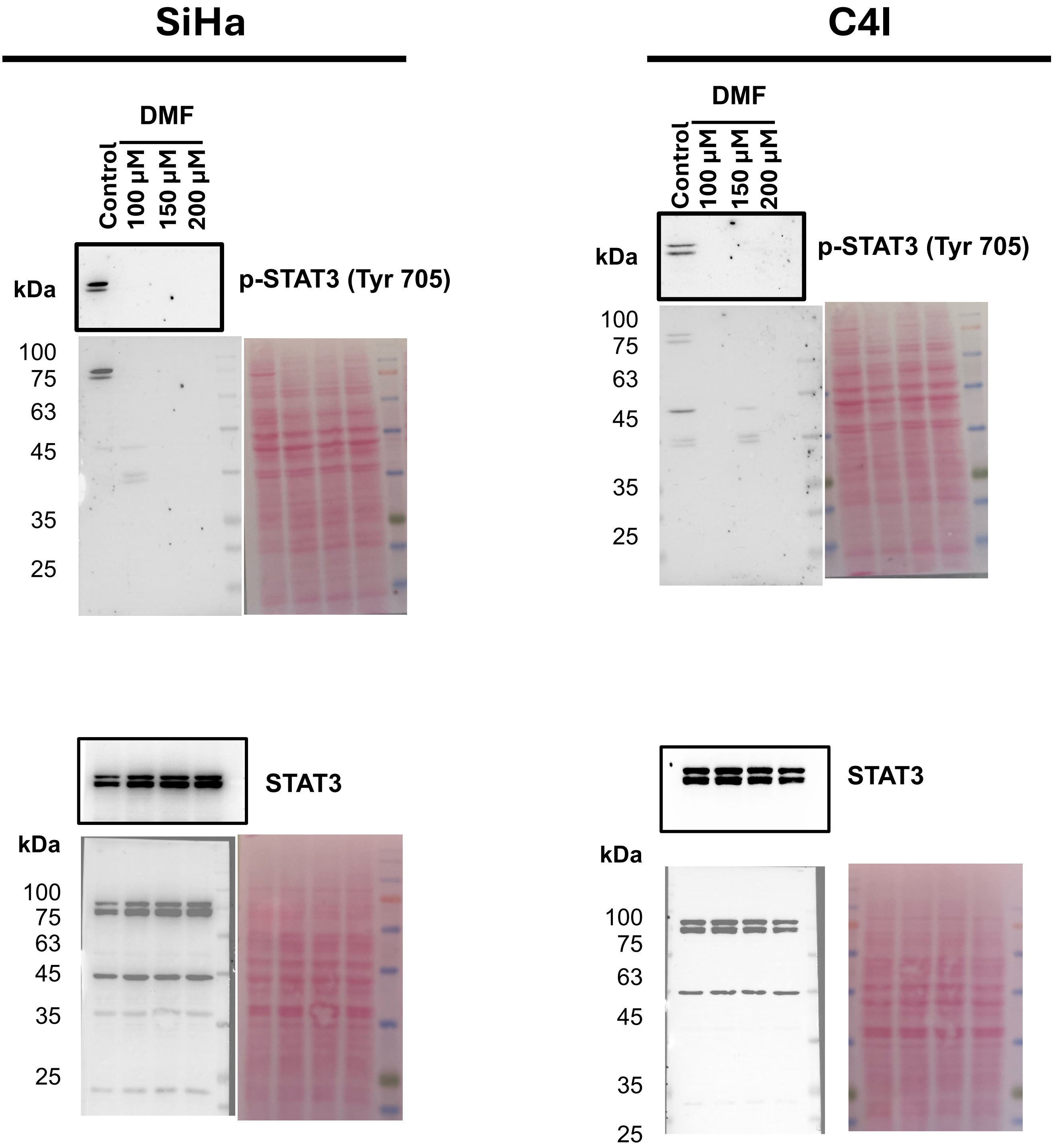

Figure S2. Effect of Dimethyl fumarate (DMF) on STAT3 signaling. Western blot analysis of p-STAT3 (Tyr 705) and total STAT3 was performed on SiHa and C4I cells upon exposure to DMF 100, 150 and 200 µM as well as to vehicle (control) for 6 hours. Ponceau S was used for loading control (n = 3).

Figure S3A

SiHa

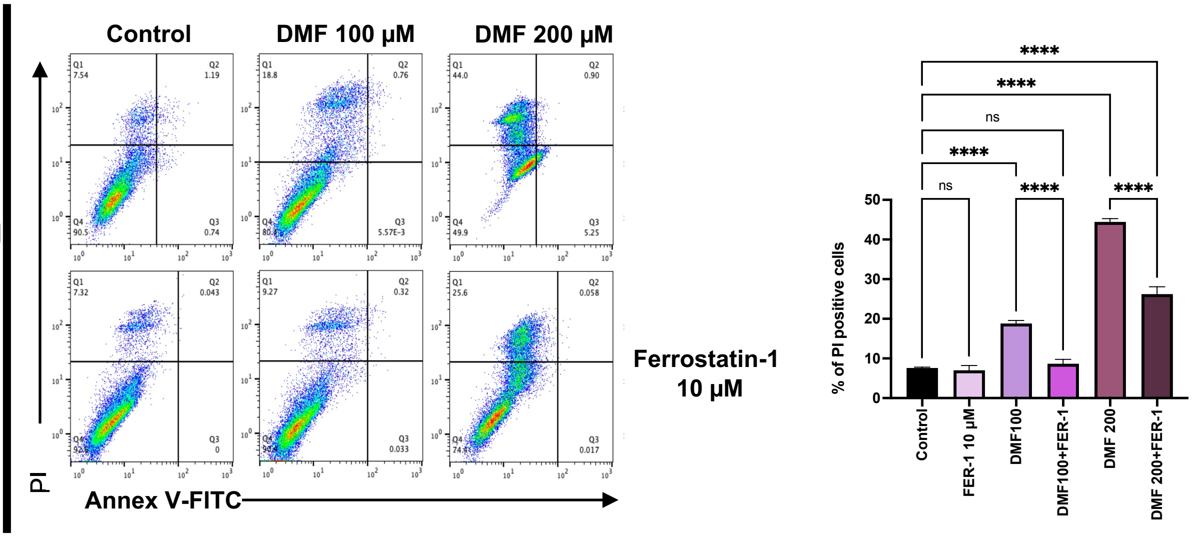

C4I

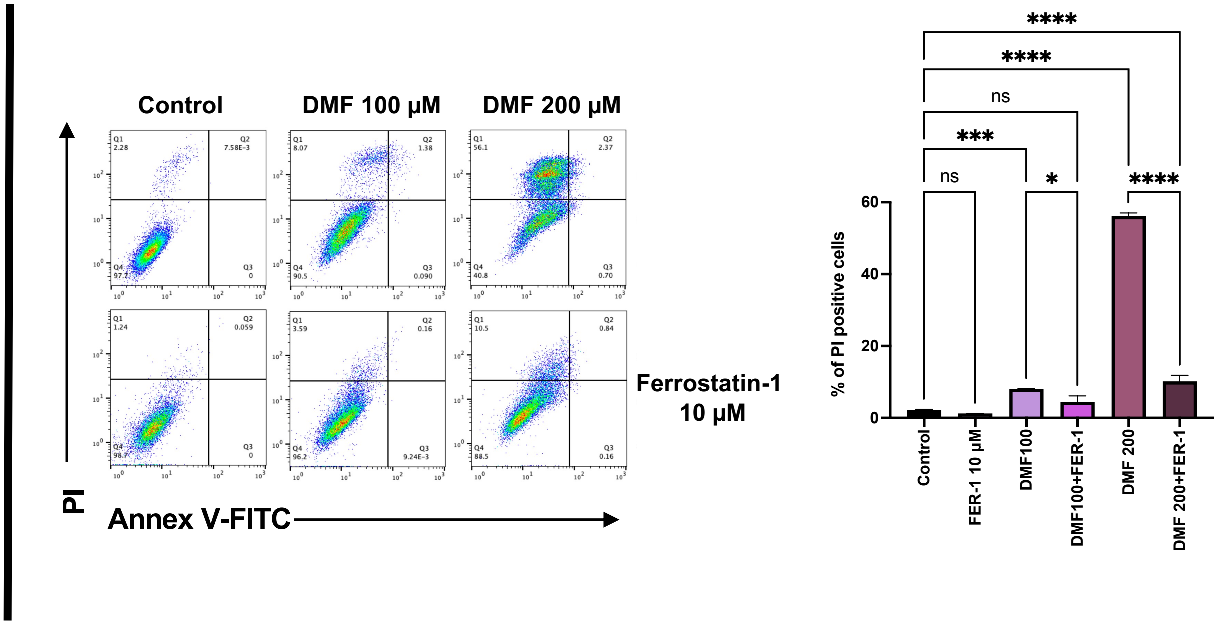

Figure S3B

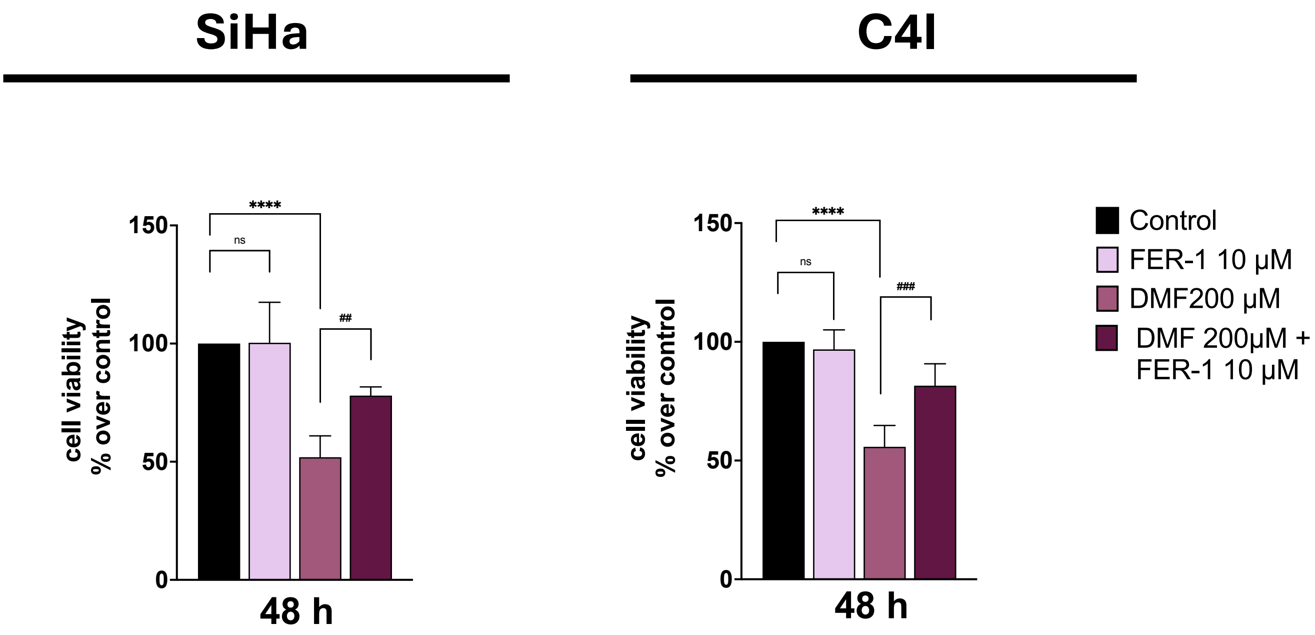

Figure S3. Dimethyl fumarate (DMF) at 200 μM induces ferroptosis in SiHa and C4I cells.

A) SiHa and C4I cells were treated with indicated doses of DMF in presence or not of Ferrostatin (FER-1) 10 μM for 72 hours. Cell death was analyzed by using Annexin-V/Propidium iodide (PI) staining followed by flow cytometry analyses; on the left representative dot plots; on the right quantification of PI positive cells. Data represent means ± SD from independent experiments (n = 3). Statistical significance was assessed by one-way ANOVA; * p≤ 0.01, *** p≤ 0.001, **** p ≤ 0.0001, ns = not statistically significant.

B) SiHa and C4I cells were treated with Ferrostatin (FER) 10 μM or DMF 200 μM or with both drugs for 48 hours. Cell viability was measured by CCK-8 assay and expressed as a percentage relative to cells treated with vehicle (control) or cells treated with DMF at 200 μM. Data represent means ± SD from independent experiments (n = 4). Statistical significance was assessed by one-way ANOVA: ## p ≤ 0.01, ### p ≤ 0.001, **** p ≤ 0.0001, ns = not statistically significant.

Figure S4

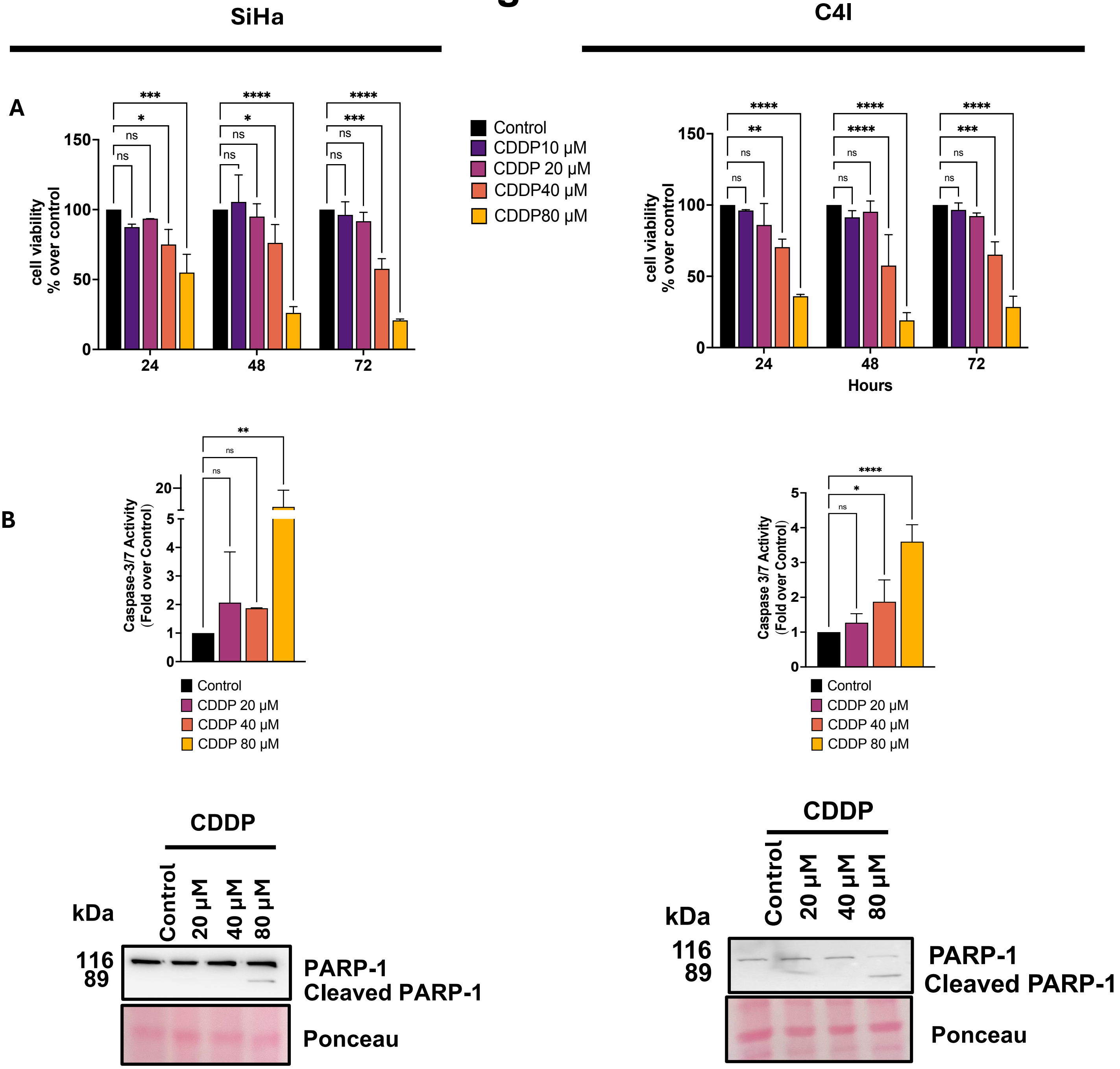

Figure S4. Dose-dependent effects of cisplatin (CDDP) on SiHa and C4I cells. A) SiHa and C4I cell viability was evaluated after exposure of cells to vehicle (control) or to CDDP at 10, 20, 40, or 80 µM for 24, 48 and 72 hours by using CCK8 assay. Data are expressed as relative percentages of optical density obtained in treated cells compared to control Data represent means ± SD from independent experiments (n = 4). Statistical significance was assessed by two-way ANOVA: * $p \le 0.05$, ** $p \le 0.01$, *** $p \le 0.001$, **** $p \le 0.0001$, ns = not statistically significant. B) Caspase 3/7 activity (upper panels) and PARP-1 cleavage (lower panels) in SiHa and C4I cells exposed to vehicle (control) or to various doses of CDDP (20, 40 or 80 µM) for 24 hours were assessed as described in “Materials and Methods”. Ponceau S was used for loading control of lysates in Western blotting experiments. Data represent means ± SD from independent experiments (n = 3). Statistical significance was assessed by one-way ANOVA: * $p \le 0.05$, ** $p \le 0.01$, **** $p \le 0.0001$, ns = not statistically significant.

Table S1. Primers used for RT-qPCR analyses

TARGET GENES	Forward (5'-3')	Reverse (5'-3')
**B2-microglubulin**	CCGTGGCCTTAGCTGTGCT	TCGGATGGATGAAACCCAGA
**CHAC1**	TTCTGGCAGGGAGACACCTT	GCCTCTCGCACATTCAGGTA
**GADD45α**	AGACCCCGGACCTGCACT	CCGGCAAAAACAAATAAGTTGACT
**p21**	GTGGCTATTTTGTCCTTGGGC	GTTCTGACATGGCGCCTGAA
**PTGS2**	GCCTGATGATTGCCCGACT	CGCTGTCTAGCCAGAGTTTCA
**SAT-1**	ACCCCTTTTACCACTGCCTG	TGCCAATCCACGGGTCATAG
**SLC7A11**	TGAAATCCCTGAACTTGCGAT	TCTGGATCCGGGCGCT
